# Nanocomposites of Titanium Dioxide and Peripherally Substituted Phthalocyanines for the Photocatalytic Degradation of Sulfamethoxazole

**DOI:** 10.3390/nano12193279

**Published:** 2022-09-21

**Authors:** Joanna Musial, Artium Belet, Dariusz T. Mlynarczyk, Michal Kryjewski, Tomasz Goslinski, Stéphanie D. Lambert, Dirk Poelman, Beata J. Stanisz

**Affiliations:** 1 Chair and Department of Pharmaceutical Chemistry, Faculty of Pharmacy, Poznan University of Medical Sciences, Grunwaldzka 6, 60-780 Poznań, Poland; 2 Department of Chemical Engineering–Nanomaterials, Catalysis, Electrochemistry, University of Liege, Building B6a, Allée du 6 Août 11, B-4000 Liège, Belgium; 3 Chair and Department of Chemical Technology of Drugs, Faculty of Pharmacy, Poznan University of Medical Sciences, Grunwaldzka 6, 60-780 Poznań, Poland; 4 Chair and Department of Inorganic and Analytical Chemistry, Faculty of Pharmacy, Poznan University of Medical Sciences, Rokietnicka 3, 60-806 Poznań, Poland; 5 LumiLab, Department of Solid State Sciences, Ghent University, Krijgslaan 281 S1, B-9000 Ghent, Belgium

**Keywords:** photocatalysis, photodegradation, phthalocyanine, sulfamethoxazole, titanium dioxide

## Abstract

Phthalocyanines (Pcs) are often used in photosensitization of titanium(IV) oxide, a commonly employed photocatalyst, as such an approach holds the promise of obtaining highly stable and efficient visible light-harvesting materials. Herein, we report on the preparation, characterization and photoactivity of a series of composites based on TiO_2_ and peripherally modified metallophthalocyanines: either tetrasulfonated or 4,4′,4′′,4′′′-tetraazaphthalocyanines, with either copper(II), nickel(II) or zinc(II) as the central metal ion. Physicochemical characterization was performed using UV-Vis diffuse reflectance spectroscopy, hydrodynamic particle-size analysis, surface-area analysis using N_2_ adsorption-desorption measurements and thermogravimetry combined with differential scanning calorimetry. The band-gap energy values were lower for the composites with peripherally modified phthalocyanines than for the commercial TiO_2_ P25 or the unsubstituted zinc(II) phthalocyanine-grafted TiO_2_. TG–DSC results confirmed that the chemical deposition, used for the preparation of Pc/TiO_2_ composites, is a simple and efficient method for TiO_2_ surface modification, as all the Pc load was successfully grafted on TiO_2_. The photocatalytic potential of the Pc/TiO_2_ materials was assessed in the photocatalytic removal of sulfamethoxazole—a commonly used antibacterial drug of emerging ecological concern. To compare the activity of the materials in different conditions, photodegradation tests were conducted both in water and in an organic medium.

## 1. Introduction

Currently, conventional wastewater treatment facilities do not ensure complete elimination of certain chemical contaminants, including pharmaceuticals, and these are thus termed persistent pollutants. This group of molecules is of particular concern as they are designed to elicit a precise biological response in living organisms [1,2,3,4,5]. More and more often detected in aqueous environments, mostly in sewage, surface water and groundwater, but also in drinking water sources, they may pose a threat to human health and the ecosystem [6,7]. Heterogeneous photocatalysis is an advanced oxidation process (AOP) that has recently emerged as a promising solution to this problem [8,9]. The main idea is to harvest energy from light to produce strong radicals in water that will chemically degrade the organic pollutants. Titanium(IV) oxide (titanium dioxide, TiO_2_) is one of the most commonly used photocatalysts, owing to its good photochemical properties: high photochemical activity, high physical and chemical stability, relatively low cost, simplicity of preparation as well as the possibility of reuse [10,11]. However, technologies based on photocatalysis have not yet been commonly applied on a large scale, mainly due to the narrow light absorption range of the materials. TiO_2_ is capable of absorbing light only from the UV region that constitutes merely a few percent of the solar irradiation [12], which limits its activation using natural sunlight. However, these shortcomings can be overcome by various modifications, for instance, grafting the surface of TiO_2_ with photoactive compounds [13,14,15,16]. One particularly interesting strategy is sensitization with photosensitizers that absorb light in the visible range, as it holds promise of efficient light-harvesting capability.

**Figure 1:**
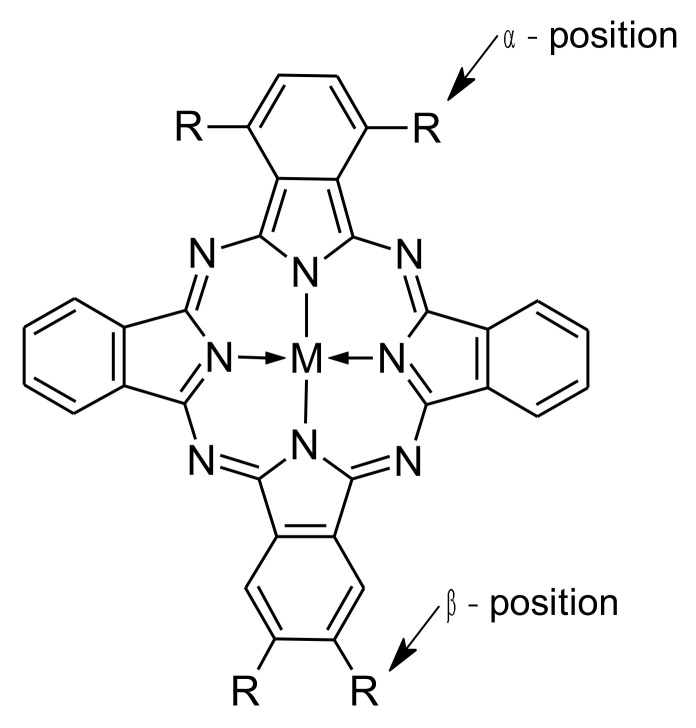
General structure of a phthalocyanine with the non-peripheral (α) and peripheral (β) substitution positions (M = metal cation or 2H).

Organic dyes are the most commonly used photosensitizers for photocatalytic water remediation. A particularly interesting group of such dyes are phthalocyanines (Pcs)—*aza*-porphyrinoids, composed of four isoindole groups linked with *aza*-methine bridges (Figure 1). Many cations may form coordination complexes with phthalocyanines. Depending on the metal ion, the molecular electronic and optical properties can be tailored to some extent [17]. Most importantly, phthalocyanines are photoactive molecules that absorb light in two main wavelength ranges, with peaks around 350 and 670 nm (Soret band and Q band, respectively), and are able to mediate reactive oxygen species (ROS) generation. This favorable absorption spectrum can enable more efficient use of solar irradiation and, therefore, minimize the external energy needed during the application of the AOPs in wastewater treatment (such as the use of a UV lamp). However, most phthalocyanines are non-soluble and prone to aggregation in aqueous solutions [18]. For these reasons, phthalocyanines are effectively used as photosensitizing grafting materials, as both the Pc and the sensitized nanoparticles benefit from such a combination [18,19,20,21,22,23,24,25].

Encouraged by our earlier results [26,27], we continued the studies on the photocatalytic activity of composites of TiO_2_ and phthalocyanines to provide a deeper insight into the structural details of the macrocycles. We tested metallophthalocyanines with different peripheral groups and central metal ions. Peripheral structure modifications can strengthen the attachment of the macrocycle on the surface of TiO_2_ by enabling chemical bond formation (instead of physisorption of the photosensitizer on the surface of the photocatalyst). Chemical anchoring is important for two main reasons: it can increase the efficiency of the charge transfer process and also provide good stability of the composites [28]. In addition, the type of metal cation in the phthalocyanine central cavity influences the light absorption spectrum, the type of the reactive oxygen species produced, aggregation of the molecules and the photobleaching phenomenon [17,29].

The present study aimed to prepare a series of phthalocyanine-sensitized TiO_2_ nanoparticles and assess their photocatalytic performance. The physicochemical properties of the as-prepared materials were determined. Particular emphasis was put on the central metal ion and the peripheral group of the phthalocyanine, as well as a detailed morphological characterization by gas sorption and thermogravimetry of the obtained materials. Furthermore, the photocatalytic application potential of the composites was tested in the degradation of sulfamethoxazole (SMX), a pharmaceutical compound of emerging ecological concern. This compound can be used as a representative benchmark molecule for drugs that can be found in the effluents of a wastewater treatment plant. The relationship between the properties of the phthalocyanine and its photosensitizing potential in a photocatalytic degradation process is discussed, paying attention to both the stability and the activity of the composites.

## 2. Materials and Methods

### 2.1. Materials and Instruments

All the solvents and reagents used for the synthesis were purchased from commercial suppliers and used without further purification. All the glassware used for chemical reactions was heated beforehand at 140 °C. All the reactions were performed in an inert gas atmosphere unless otherwise stated. *Radleys Heat-On*™ heating system was used for stirring (and heating where appropriate) the reaction mixtures. For the reactions conducted under microwave irradiation, a microwave reactor *Monowave 400* (Anton Paar GmbH, Graz, Austria) was used.

**Figure 2:**
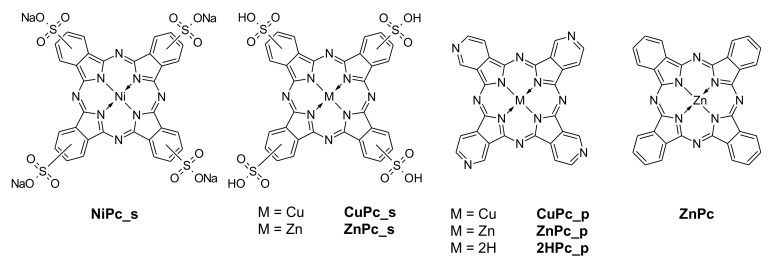
Chemical structures of the phthalocyanine derivatives used in this study.

Commercially available phthalocyanines–nickel(II) phthalocyanine-tetrasulfonic acid (**NiPc_s**), copper(II) 4,4′,4′′,4′′′-tetraazaphthalocyanine (**CuPc_p**), copper(II) phthalocyanine-tetrasulfonic acid (**CuPc_s**), and zinc(II) phthalocyanine (**ZnPc**) were purchased from Sigma-Aldrich and used without further purification or any modifications. Other macrocyclic compounds were synthesized according to the literature methods: 4,4′,4′′,4′′′-tetraazaphthalocyanine (**2HPc_p**) [30], zinc(II) 4,4′,4′′,4′′′-tetraazaphthalocyanine (**ZnPc_p**) [31], and zinc(II) phthalocyanine-tetrasulfonic acid (**ZnPc_s**) [32]. Any changes in the synthetic procedures are given in the Supplementary Materials (Schemes S1, S2 and S3). Chemical structures of the phthalocyanine derivatives used in this study are presented in Figure 2.

### 2.2. Preparation of Photocatalytic Materials

**Figure 3:**
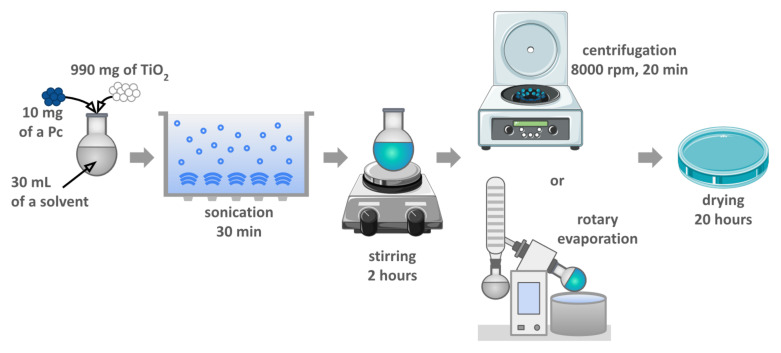
Schematic representation of the preparation of the photocatalytic materials by the chemical deposition method.

The photocatalytic materials were prepared according to the chemical deposition method described previously [26,27,33]. The applied protocol was designed for theoretical 1% (*w*/*w*) phthalocyanine grafting. All the prepared materials were based on commercially available TiO_2_ P25 (Aeroxide, 21 nm primary particle size, purchased from Sigma-Aldrich). The overview of the procedure is illustrated in Figure 3, and all the modifications are presented in Table 1.

The first step consisted in dissolving 10 mg of the Pc using 30 mL of the solvent (indicated in Table 1) in a round-bottom flask. Next, 990 mg of TiO_2_ was added, the suspension was sonicated for 30 min and then stirred for 2 h at room temperature. The solid material was thereafter separated by centrifugation (8000 rpm, 20 min), washed with ethanol, and separated by centrifugation again (8000 rpm, 20 min, repeated three times). In the case of ZnPc deposition, the solvent was removed by using a rotary evaporator. In all cases, the resulting blue solid was dried in air overnight to remove potential residual solvents.

### 2.3. Characterization of Photocatalytic Materials

#### 2.3.1. UV-Vis Diffuse Reflectance Spectroscopy (DRS)–Band Gap Determination

The band gaps of the photocatalytic materials were determined using diffuse reflectance spectroscopy measurements in the region of 250–800 nm using a Lambda 1050 S UV–Vis–NIR spectrophotometer (Perkin Elmer) equipped with a Spectralon-coated integrating sphere. The recorded diffuse reflectance spectra ($R_{\mathrm{sample}}$) were transformed into absorbance spectra using the Kubelka–Munk equation [33]:

(1) $$F\left(R_{\infty }\right)=\frac{{\left(1-R_{\infty }\right)}^{2}}{2R_{\infty }}$$

where $R_{\infty }$ is defined as $R_{\infty }=\frac{R_{\mathrm{sample}}}{R_{\mathrm{reference}}}$ with $R_{\mathrm{reference}}$ being the diffuse reflectance spectrum measured for the Spectralon reference. Spectra were normalized in intensity to 1 by dividing each spectrum by its maximum. Applying the Tauc plot method [34], the following equation:

(2) $${\left(F\left(R_{\infty }\right)h\nu \right)}^{1/m}=C\left(h\nu -E_{g}\right)$$

(where C is a constant and m is a constant that depends on the optical transition mode), was used to obtain the direct and indirect optical band gap values, $E_{g,\mathrm{direct}}\;$(eV) and $E_{g,\mathrm{indirect}}\;$(eV), by plotting ${\left(F\left(R_{\infty }\right)h\nu \right)}^{2}$ and ${\left(F\left(R_{\infty }\right)h\nu \right)}^{1/2}$, respectively, as a function of the photon energy hν and by determining the intersection of the linear part of the curve and the energy axis.

#### 2.3.2. Hydrodynamic Particle Size

The nanoparticle size distribution in aqueous suspension was evaluated by nanoparticle tracking analysis (NTA) using a *Malvern Panalytical* (Malvern, UK) *NanoSight LM10* instrument equipped with a sCMOS camera (405 nm laser) and 3.2 Dev Build 3.2.16 software. The nanomaterial dispersions were diluted with deionized water to reach the operating range of nanoparticle concentration. The sample chamber temperature was set and maintained at 25.0 ± 0.1 °C, and the syringe pump infusion rate was set to 200. Three movies of 30 s were recorded for each sample.

#### 2.3.3. Morphological Analysis by the N_2_ Adsorption-Desorption Method

A detailed morphological characterization using the N_2_ adsorption-desorption method at 77 K was performed using the *ASAP™ 2420 system* (Micromeritics^®^, Norcross, GA, USA). Prior to the analysis, samples were outgassed at 200 °C for 16 h to remove any undesirable adsorbed species. Information such as specific surface area, external specific surface area, porous volume, porosity, radius and relative average number of contacts (if a spherical geometry is supposed) was retrieved from these analyses.

#### 2.3.4. TG-DSC

Thermogravimetric (TG) analysis coupled with differential scanning calorimetry (DSC) was performed using a *SETARAM^®^ Sensys Evo 3D* TG–DSC with a platinum crucible. Platinum was chosen instead of the commonly used alumina to avoid side reactions as well as diffusion phenomena that could occur when metallic oxides are put together at higher temperatures. Samples of a given mass were heated from room temperature to 800 °C at a rate of 2 °C/min. Grafting yields were calculated from the mass loss. DSC plots provided insights into the chemistry of the Pcs and Pcs immobilized on TiO_2_.

### 2.4. Photochemical Studies

#### 2.4.1. Set-Up of the Photocatalytic Experiment in an Organic Solvent

**Figure 4:**
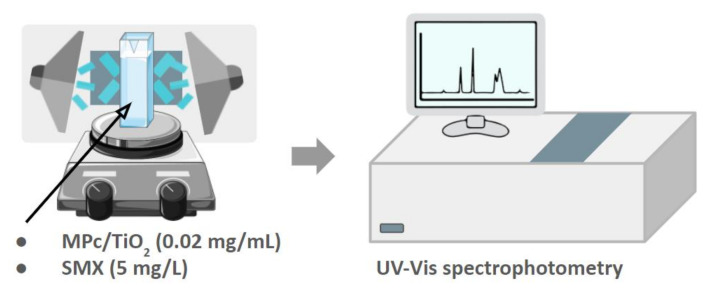
Set-up of the photocatalytic experiment in DMF.

The photodegradation experiments in *N,N*-dimethylformamide (DMF) were carried out in a reactor consisting of a UV-transparent cuvette placed on a magnetic stirrer in the middle and surrounded by three LED lamps, either at 365 (UV) or 665 nm (R). The light intensity was set to 20 mW/cm^2^ and monitored with an *RD 0.2*/*2* radiometer (Optel, Opole, Poland) (Figure 4). The cuvette contained a total volume of 3.0 mL, composed of 0.75 mL of SMX solution (20 mg/L), 0.75 mL of a photocatalyst suspension (0.08 mg/mL) and 1.5 mL of DMF. SMX solution was obtained by dilution from a stock solution prepared by dissolving 100 mg of SMX in 5.0 mL of DMF. Similarly, photocatalyst suspensions were obtained by dilution from a stock solution prepared by suspending 6.0 mg of a photocatalytic composite in 15.0 mL of DMF. Each mixture of SMX and a photocatalyst was irradiated for 10 min while being constantly stirred. The absorbance values at 267 nm, corresponding to the SMX absorption maximum, were measured using a *Jasco V–770* UV–Vis spectrophotometer (Jasco, Tokyo, Japan) at the following times: 0, 2, 4, 6, 8, and 10 min after starting illumination.

#### 2.4.2. Set-Up of the Photocatalytic Experiment in Water

**Figure 5:**
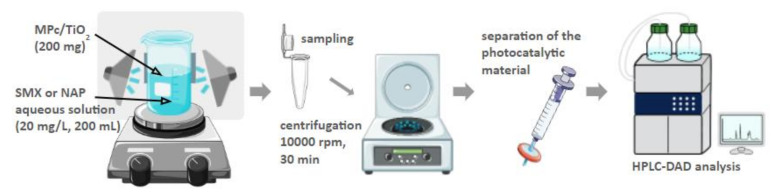
Schematic representation of the set-up and general procedure of the photocatalytic experiment in water.

The photodegradation experiments in water were carried out in a reactor consisting of a beaker (transparent for light over 200 nm) placed on a magnetic stirrer and surrounded by three LED lamps, either UV or R, as described before (Figure 5). The beaker contained 200 mL of SMX solution at 20 mg/L. This solution was prepared each time by dilution from a stock solution, prepared beforehand by dissolving 100.0 mg of SMX in 5.0 mL of methanol. Then, 200 mg of photocatalyst was added, and the resulting mixture was sonicated in the dark for 5 min.

Next, the mixture was stirred for 30 min without irradiation to reach the adsorption-desorption equilibrium [35,36,37]. After the ‘dark step’, either UV or R LED lamps were turned on. The mixtures were irradiated for 8 to 24 h while being constantly stirred. Samples of 2 mL were taken in triplicate at the following time points: −0.5, 0, 2, 4, 6, 8, and 24 h. Collected samples were centrifuged at 10,000 rpm for 30 min. Prior to the HPLC analysis, the samples were additionally filtered through 0.2 μm PTFE syringe filters.

SMX concentrations were measured using an HPLC instrument equipped with a diode array detector (DAD) (Agilent 1100 HPLC, PerkinElmer, Waltham, MA, USA), operating at a wavelength of 267 nm, and an Agilent Eclipse XDB C18 analytical column (150 × 4.6 mm, 5 µm). The temperature of the column was set at 30 °C. The flow rate was set at 1.0 mL/min, and the injected sample volume was 5 μL. The mobile phase consisted of methanol and water, 50:50 (*v*/*v*).

## 3. Results and Discussion

### 3.1. Preparation of Photocatalytic Materials

**Figure 6:**
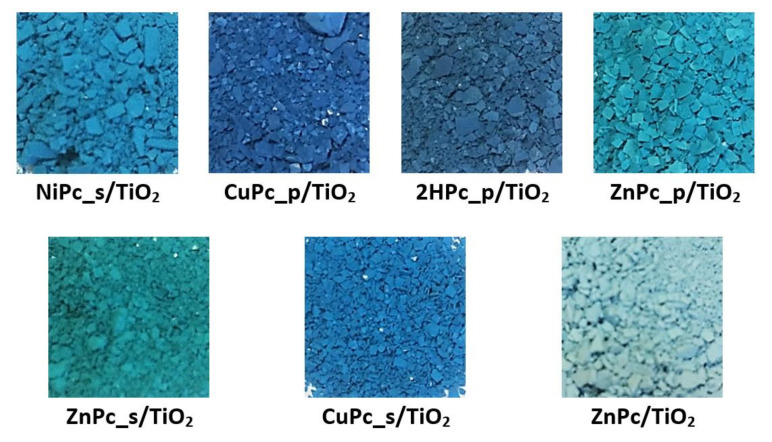
Photographs of the obtained photocatalytic composites.

TiO_2_ P25, a mixture of two polymorphs—anatase and rutile—was chosen as a support for macrocyclic compounds. Rutile is generally considered less active, yet more stable, than pure anatase in photocatalytic applications [38,39]. However, data from several studies show that thanks to a better electron-hole pair separation, the mixed-phase TiO_2_ appears to be more active than either pure anatase or pure rutile [40,41,42]. The nanomaterials were prepared using a simple chemical deposition procedure, yielding a series of photocatalysts, each containing a theoretical 1% (*w*/*w*) of one of the following phthalocyanines deposited on the surface: **NiPc_s** (NiPc_s/TiO_2_), **CuPc_p** (CuPc_p/TiO_2_), **2HPc_p** (2HPc_p/TiO_2_), **ZnPc_p** (ZnPc_p/TiO_2_), **ZnPc_s** (ZnPc_s/TiO_2_), **CuPc_s** (CuPc_s/TiO_2_), and **ZnPc** (ZnPc/TiO_2_) (Figure 6).

According to our previous studies, the chemical deposition procedure does not alter the crystallinity of the TiO_2_ nanoparticles [26]. During the deposition process, it was noticed that to attach Pcs on the surface of TiO_2_ efficiently, the pH of the suspension must be adjusted to 3–4. After centrifugation, which followed 2 h of stirring, it was observed that the solution became colorless and transparent, which indicated that the phthalocyanine was efficiently deposited on the surface of TiO_2_. This observation was later confirmed by calculating the Pc content via TG-DSC measurements (Section 3.2.4).

**Figure 7:**
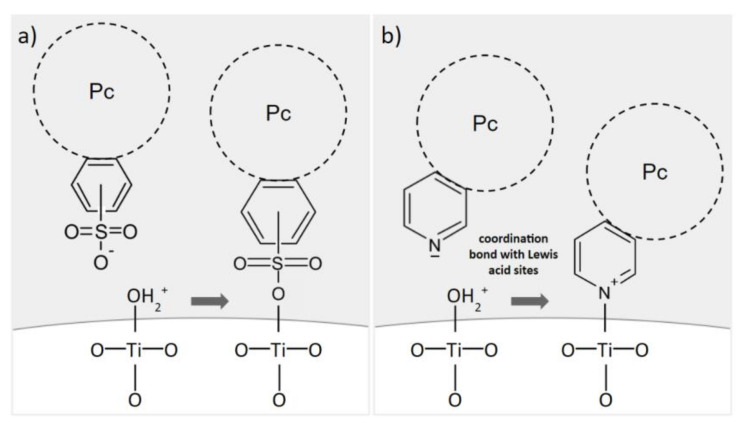
Proposed anchoring modes for (**a**) sulfonyl and (**b**) pyridyl peripheral groups of phthalocyanines on TiO_2_ surface during the chemical deposition process below the TiO_2_ point of zero charge (pH = 3–4). Based on [28,45,53].

The pH range of 3–4 is below the point of zero charge of TiO_2_ [43]. Therefore, a certain amount of positively charged hydroxyl groups on its surface favors the formation of physicochemical bonds with negatively charged, peripheral groups of Pcs. Figure 7 presents possible anchoring modes for both types of peripherally modified Pcs used in the present study. In the case of tetrasulfonated Pcs, in the pH range of 3–4 and considering the pKa of the sulfonic groups being negative, the main species are all negatively charged sulfonates, and they would form a covalent bond with superficial hydroxyl groups [44,45,46]. Sulfonic acid groups have been reported to improve the performance of TiO_2_-based photocatalysts [47]. The existing literature shows that for sulfonic acid groups, monodentate, bidentate and tridentate binding modes are possible [48,49,50]. In the case of tetraazaphthalocyanines, a coordination bond between the electron-acceptor sites of titania and the non-shared electron pair of nitrogen in the pyridyl ring can be formed [51,52,53,54,55]. Our observations are in accordance with the work of Mathew et al., who reported that the protonation of each pyridyl nitrogen in 5,10,15,20-tetra(4-pyridyl)porphyrin derivative proceeds step by step, in four equilibrium reactions, with pKa values estimated to be: 5.3, 4.7, 2.2 and 1.2, respectively [56]. Therefore, in the considered pH range of 3–4, at least two of the four pyridyl nitrogens are protonated. As a consequence, some of the pyridyl rings within the same Pc are in their basic form, which allows the mechanism shown in Figure 7. Figure S1 shows the distribution of protonated pyridyl species, according to the aforementioned pKa values [57]. Moreover, we observed that acidic conditions were necessary to solubilize the tetraaza-Pc, yet the excess of HCl hampered its deposition on the surface of TiO_2_–in such case, after the deposition, the precipitating solid TiO_2_ remained white, while the liquid was dark blue. Thus, we can state that a pH of 3–4 provided balanced conditions, favoring both the solubilization of the tetraazaphthalocyanine and the formation of a chemical bond between the pyridyl group and TiO_2_.

Unfortunately, due to the low content of the phthalocyanine (~1%), the formed bond signals could not be directly confirmed by ATR-FTIR analyses, as they overlap with other functional groups in the materials (ester bonds with Ti=O, Ti–OH and H_2_O; pyridynium groups with pyridyl groups). The indirect evidence of the chemical bonding between the Pc and TiO_2_ surface is that it was impossible to wash out the Pcs with inorganic (both water based HCl or NaOH in case of Pc_p and Pc_s, respectively) or organic (methanol, dichloromethane, *N,N*-dimethylformamide, dimethylsulfoxide, tetrahydrofuran) solvent. After addition of a solvent, the suspension was shaken, sonicated and centrifuged. The resulting supernatants were monitored by UV-Vis, which showed no traces of Pcs.

### 3.2. Characterisation of Photocatalytic Materials

#### 3.2.1. UV Diffuse Reflectance Spectroscopy (DRS)—Band Gap Determination

The UV-DRS measurements showed that grafting the TiO_2_ surface with peripherally-modified Pcs affected the resulting absorption spectra (Figure S2). Compared to bare TiO_2_, which absorbs light only in the UV region, the grafted photocatalysts clearly display two regions of absorption: UV and visible (red) region (550–750 nm), corresponding to the Soret and Q bands, respectively. The only exception is the absorption spectrum for ZnPc (without peripheral modifications), which shows strong UV absorption and a relatively weak visible light absorption. Therefore, DRS results transformed into normalized Kubelka–Munk absorption functions confirmed that the obtained materials are capable of absorbing light in the irradiation range of the photocatalytic tests: UV (λ_max_ = 365 nm) or R (λ_max_ = 665 nm).

**Figure 8:**
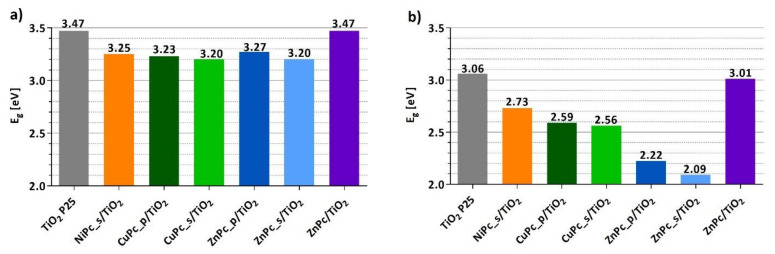
Band-gap energy values (E_g_) of each photocatalytic material calculated from DRS measurements using the Kubelka–Munk equation and the Tauc plot method, assuming a direct transition (**a**) or an indirect transition (**b**).

Figure 8 presents the comparison of the band-gap energy values ($E_{g}$) calculated for each of the photocatalytic materials, assuming the direct as well as the indirect electronic transitions from the valence band to the conducting band (Table S1). Grafting TiO_2_ with peripherally-modified phthalocyanines reduced the $E_{g}$ of the photocatalytic material. The results are consistent with the literature [58,59,60]. There is a clear difference between the $E_{g}$ of bare TiO_2_ and the $E_{g}$ of composites of peripherally-modified Pcs and TiO_2_; however, these composites express similar $E_{g}$ values. Interestingly, the $E_{g}$ for unmodified ZnPc deposited on TiO_2_ is equal to that of bare TiO_2_, whereas for other materials containing functionalized derivatives of zinc(II) phthalocyanine, either with peripheral pyridyl (ZnPc_p) or sulfonyl (ZnPc_s) groups, the $E_{g}$ value is significantly lower.

A graphical representation of the normalized Kubelka–Munk function (Equation (1)) for each photocatalytic material can be found in the Supporting Information (Figures S3 and S4).

#### 3.2.2. Particle Size

The mean hydrodynamic diameter of each composite was determined using nanoparticle tracking analysis. This technique, similar to dynamic light scattering, measures the hydrodynamic diameter of the particles, which is higher than the geometric diameter because it includes the electrical double layer on the particle surface in a liquid medium. The results indicate that each of the tested materials, including unmodified TiO_2_, the particles are prone to agglomeration in a liquid medium (Table 2).

#### 3.2.3. N_2_ Sorption Analysis

The specific surface area (S_BET_) was calculated using the multilayer theory of Brunauer–Emmett-–Teller on the adsorption curve of the N_2_ sorption experiment at 77 K. The external specific surface (S_EXT_), as well as the microporous volume, were calculated by the t-plot method (adsorption curve), using the Harkins and Jura statistical thickness equation extrapolated in the linear range, corresponding to the theoretical formation of a N_2_ monolayer. The arbitrary choice of the linearity range affects the calculated values of S_EXT_ and the microporous volume. Although this is still under debate in literature [61], a range between t = 0.35 nm and t = 0.50 nm was chosen for all materials. The obtained values were found negligible (Table 3), thus all the microporous volumes can be considered approximately 0 cm³/g. The apparent negative values of micropores’ volume are the result of the chosen linear range. Further comparison showed no significant differences neither in S_BET_ nor S_EXT_ values among the photocatalytic composites (Table 3). Hence, the obtained results support the assumption that the prepared materials are non-porous.

Furthermore, assuming a spherical geometry for the dense non-porous particles, it is possible to calculate a corresponding diameter, or particle size, from S_EXT_ via the following equation:

(3) $$D=\frac{6}{\rho_{{\mathrm{TiO}}_{2}}\times S_{\mathrm{EXT}}\times 10^{-3}}$$

where D is expressed in nm, $\rho_{{\mathrm{TiO}}_{2}}$ is the density of polycrystalline TiO_2_ in g/cm³ (a value of 4.26 was considered, as provided by Sigma-Aldrich, Saint Louis, MO, USA), and S_EXT_ is in m²/g. The calculated particle sizes are reported in Table 3. For all MPc/TiO_2_ materials, we observed a slight increase in the particle size compared to the unmodified TiO_2_, in agreement with a system seen as large molecules covering the surface of rigid spheres. Still, a discrepancy is observed between the unmodified TiO_2_ particle size calculated here (25.4 nm) and the one provided by Sigma-Aldrich (21 nm). The difference may stem from different characterization techniques used to determine this value or the simplifications used in the provided calculations.

**Figure 9:**
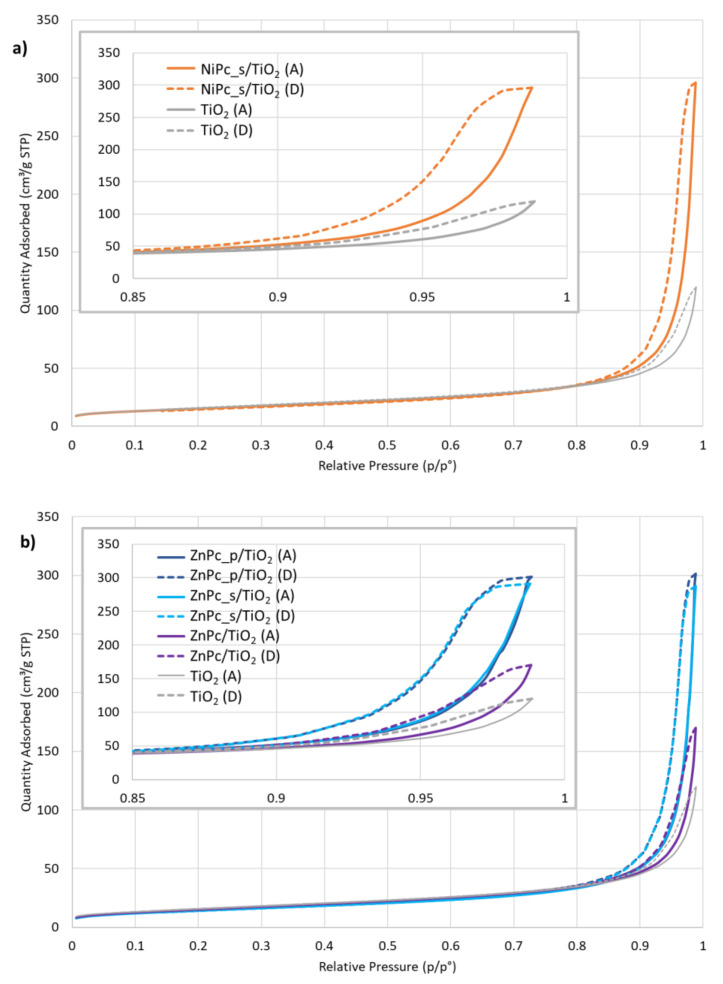

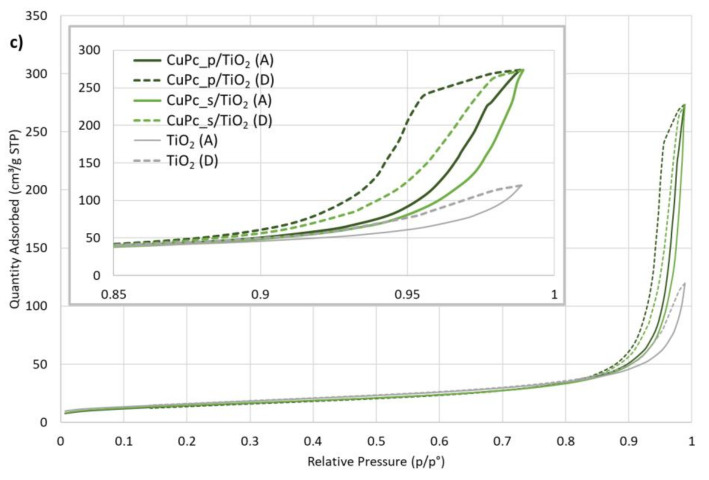
Molecular nitrogen (N_2_) physisorption isotherms at 77 K for: (**a**) NiPc_s/TiO_2_, (**b**) ZnPc_s/TiO_2_, ZnPc_p/TiO_2_ and ZnPc/TiO_2_, (**c**) CuPc_p/TiO_2_ and CuPc_s/TiO_2_; A—adsorption isotherm, D—desorption isotherm.

Figure 9 shows N_2_ physisorption isotherms for the photocatalytic materials, compared each time with bare TiO_2_. According to the IUPAC classification of physisorption isotherms [62], the shape of the isotherms resembles type II and type IV isotherms, the latter type being considered because of the presence of a hysteresis. Hysteresis loops are associated with a capillary condensation phenomenon inside mesopores. However, we stated that our material is non-porous and no sign of mesoporosity has been observed on the t-plot (Figure S5). In accordance with the work of Gommes et al. [63], we understand that mesoporous zones can exist in between rigid spherical particles when the latter agglomerate. Moreover, the authors show that the higher the average number of contacts between spheres (N_C_), the higher the amount of N_2_ that can be adsorbed (Figure 5 in [63]). The adsorbed amount of N_2_ is systematically higher for Pc-grafted TiO_2_ particles than for unmodified TiO_2_. Similarly, in the case of zinc (II) phthalocyanines, these values are higher for the composites containing peripherally-modified Pc, than for unmodified ZnPc/TiO_2_ (Figure 9b). According to Gommes et al., these observations can be qualitatively analysed with the N_C_ concept, even if without a standard isotherm for a flat non-porous TiO_2_, it was impossible to calculate N_C_ directly. The presence of Pcs at the surface of TiO_2_ tends to unambiguously increase N_C_, compared to the bare TiO_2_: Pcs seem to act as local glue drops, due to a presumed Van der Waals interaction occurring from both sides of Pcs and joining the two spheres together. Figure 9b shows that this effect is even more pronounced when Pcs are modified with peripheral groups, which can be explained by assuming that among the four peripheral groups, some are attached to one sphere, while the others, initially free, are attached to the other one. Altogether, these analyses corroborate the agglomerates observed during the synthesis and the large distributions obtained from mean hydrodynamic diameter measurements.

#### 3.2.4. TG-DSC

**Figure 10:**
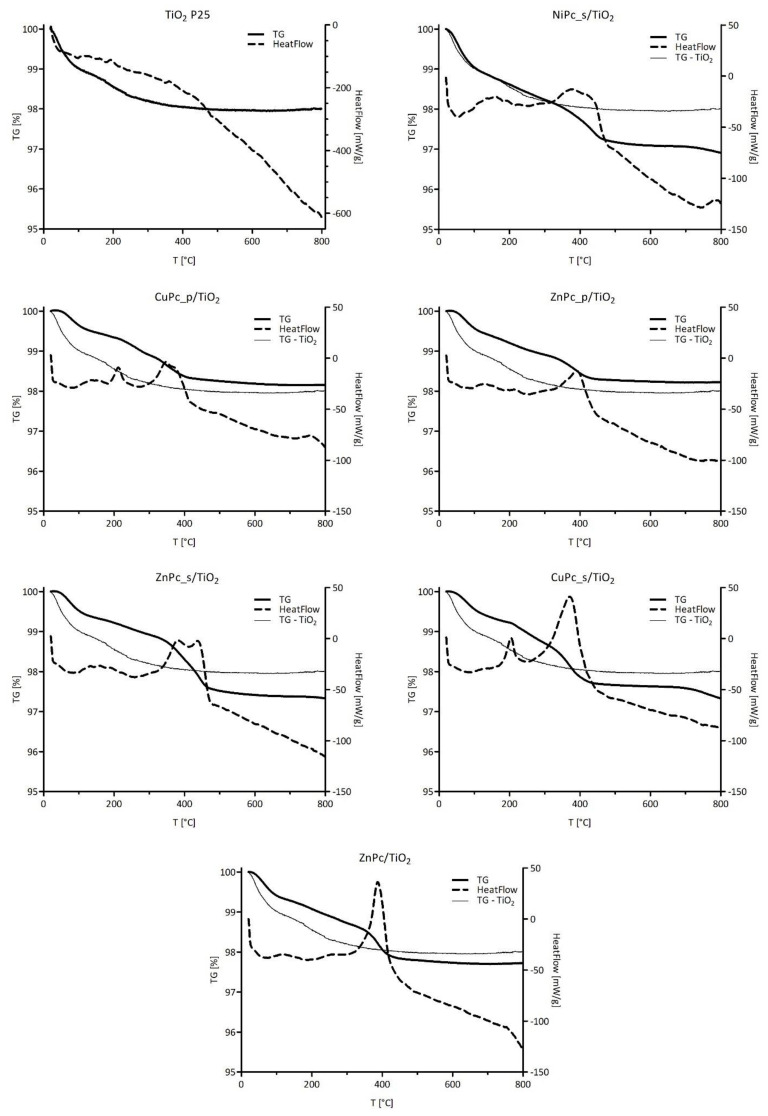
TG–DSC of prepared photocatalytic composites: TG and HeatFlow data.

The thermal stability of the materials and the amount of phthalocyanine deposited on the surface of TiO_2_ were analyzed by using TG-DSC. As shown in Figure 10, all the nanocomposites display quite similar behavior during heating. A slight mass loss, which occurs up to 150 °C is attributed to water evaporation, while the mass loss of interest occurs between 200 °C and 450 °C. The mass loss values and the corresponding temperature ranges for each nanocomposite are summarized in Table 4. For further details about our considerations for temperature values, please refer to the Supporting Information, where first derivatives of TG signals guided our choices (Figure S6). The obtained results confirmed the close theoretical 1% (*w*/*w*) Pc content in each photocatalytic material. This proves that during the chemical deposition process, all the weighed Pc was successfully grafted on the surface of TiO_2_. In the case of bare TiO_2_, 1.9% of mass loss is observed, which at first seems high, compared to the Pc-grafted TiO_2_ materials (Figure 10) [64,65,66]. However, a closer look at the mass loss up to 150 °C suggests that bare TiO_2_ can adsorb more water than Pc-grafted TiO_2_ due to its high surface hydrophilicity. This explains why, in some cases in Figure 10, the bare TiO_2_ TG curve goes below the Pc-grafted material one, even though one expects the former being above the latter. Therefore, we conclude that the hydrophilicity of the Pc-grafted materials is lower because of the lesser availability of hydrophilic sites on the surface; those sites are mainly surficial titanol groups, as well as superficial oxygen atoms from a metalloxane (Ti–O–Ti) group. The only exception seems to be NiPc_s/TiO_2_, which is able to adsorb the same amount of water as bare TiO_2_. One way to explain this observation is that Pcs, with their planar structure, cover the surface of the particle (Van der Waals interactions), instead of being loosely attached by one ending group, which results in a decrease of available hydrophilic sites.

**Figure 11:**
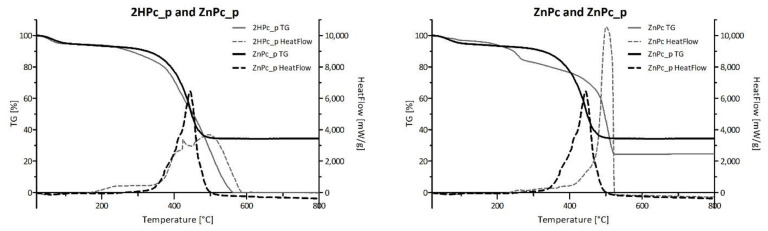

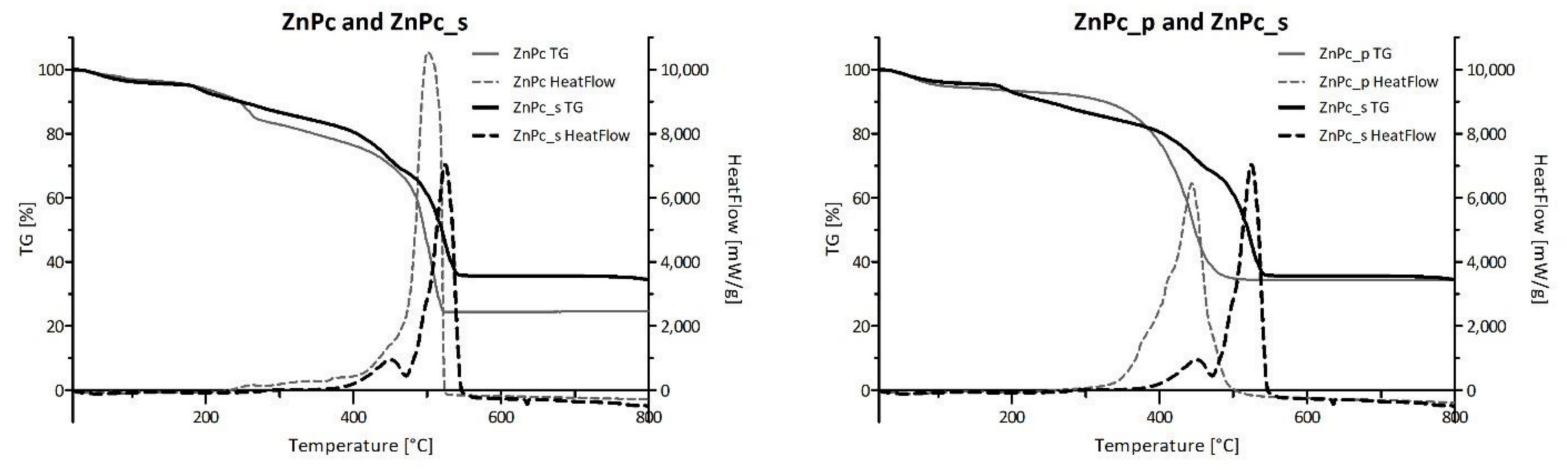
Comparison of the TG and HeatFlow curves between neat phthalocyanines. TG-DSC of each phthalocyanine presented separately is shown in Figures S7 and S8 (TG, HeatFlow and dTG data).

Figure 11 presents the comparison of TG-DSC results conducted for four neat phthalocyanines: 2HPc_p, ZnPc_p, ZnPc_s and ZnPc. Contrary to zinc(II) phthalocyanines, 2HPc_p was completely decomposed due to its purely organic structure. In the case of each of the three tested zinc(II) phthalocyanines, regardless of its peripheric group, the final mass loss was equal to 66%, 65% and 75% for ZnPc_p, ZnPc_s and ZnPc, respectively. These results are consistent with the literature and indicate that zinc compounds, which do not undergo further degradation in the tested temperature range, are formed [67,68]. Interestingly, when ZnPc and ZnPc_p are compared, a shift of the HeatFlow peak towards lower temperature can be observed. As the heat needed for the combustion of both Pcs is the same (Table 5), we can state that the shift is a result of the presence of nitrogen atoms in the pyridyl ring, which most certainly imposes a different reactional pathway than during the combustion of the unsubstituted ZnPc. In contrast, the graphical comparison between ZnPc and ZnPc_s, as well as data in Table 5 show that the heat needed for the combustion of the ZnPc_s is lower than for ZnPc (Q_Δm_ = 9778.00 J/g and 13,100 J/g for ZnPc_s and ZnPc, respectively). This finding is rather unexpected, as the addition of peripheral groups to the macrocyclic structure should require more energy input throughout the combustion process. However, it is possible that the oxygen atoms in sulfonic groups facilitate the process, reducing the heat needed for combustion.

**Figure 12:**
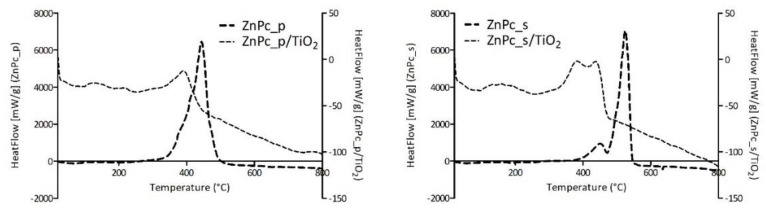

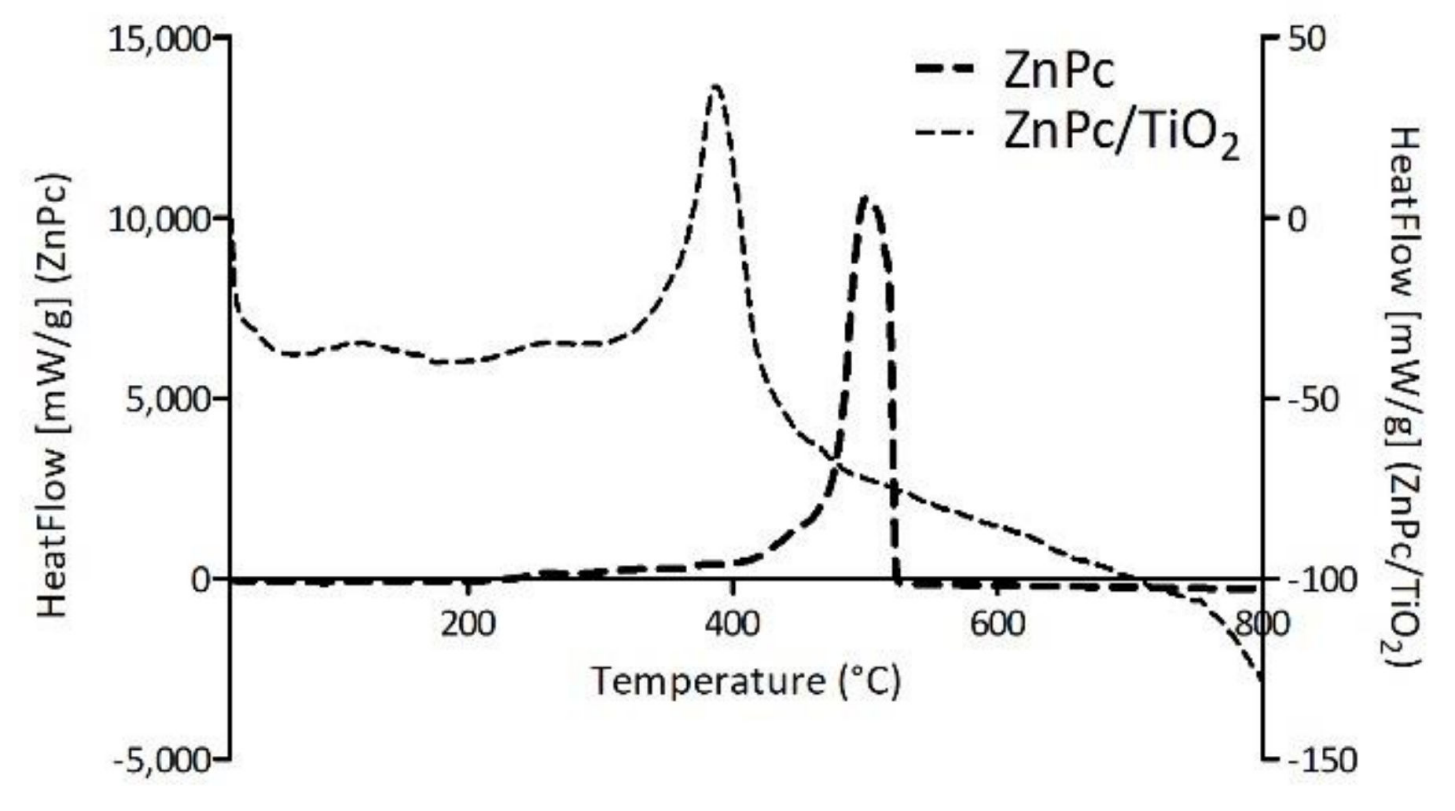
Comparison of the HeatFlow curves for neat phthalocyanines and phthalocyanine-grafted TiO_2_.

Furthermore, HeatFlow values obtained for each photocatalytic composite of TiO_2_ and zinc(II) phthalocyanine derivative and the corresponding neat Pc were set together in Figure 12. A shift of the HeatFlow peak for the neat Pc to higher temperatures can be observed when compared with the HeatFlow peak for Pc/TiO_2_ composites. This results from the adsorption of the Pc on TiO_2_, wherein the Pc on the surface of the material is more prone to combustion. It is generally acknowledged that combustion reactions occur via mechanisms involving, among others, hydroxyl radicals. We can assume that the proximity of Pcs to superficial hydroxyl groups of TiO_2_ facilitates the oxidation reactions. Hence, the shift to lower temperatures is understandable since lower thermal energy is required for those radicals to be formed from titanol groups than from oxygen species.

Moreover, the heat needed for the combustion of the phthalocyanine content in the photocatalytic composite was calculated and compared with the values obtained for the neat phthalocyanine. The results presented in Table 5 show that the heat values needed for the combustion of the Pc deposited on TiO_2_ nanoparticles are in the same order of magnitude as the values obtained for the neat Pc.

### 3.3. Photochemical Studies

The photocatalytic potential of the Pc-TiO_2_ nanocomposites was further assessed in drug degradation experiments. Sulfamethoxazole, a bacteriostatic sulfonamide compound, was chosen for the photodegradation tests as a benchmark contaminant often detected in wastewater effluents. Moreover, its elimination from the environment is of particular importance, as the dissemination of antimicrobials contributes to the progression of antibiotic microbial resistance [69,70,71].

The photocatalytic experiments were conducted in two different media—*N,N*-dimethylformamide and water—as the environment of the photocatalytic reaction is known to influence the type of the produced ROS and the aggregation tendency of metalloporphyrinoids [17]. In general, the interaction between the excited photocatalyst and oxygen may occur via type I or type II reactions. As a result of type I reactions, consisting in electron transfer, hydroxyl radicals (^•^OH), hydrogen peroxide (H_2_O_2_) and superoxide anion (^−^O^•^_2_) are produced. Type II reactions generate singlet oxygen (^1^O_2_), which has a very short lifetime in water [72]. Therefore, comparing the efficiencies of the photocatalysts in two different solvents was necessary to describe photochemical mechanisms that may drive the degradation process. Other factors that may affect the photodegradation performance include the linkage mode between the photosensitizer and the photocatalyst, the metal ion coordinated to the photosensitizer and the wavelength of light used for activation [14]. Therefore, for a clear comparison between TiO_2_ nanoparticles grafted with different phthalocyanines, distilled water was used to limit the number of variables that might have an additional impact on the photocatalytic process. The temperature of the mixture during the experiment did not exceed 30 °C. It should be noted that due to the analytical conditions (such as the need to use UV-Vis spectrophotometry to quickly analyze the samples, which required dilution of the photocatalyst suspension, as well as the adjustment of the absorbance values), the photocatalyst-to-SMX ratio in the photodegradation experiments in DMF was different than in the tests in water. The experiments, either in DMF or water, were carried out separately under the UV- (365 nm) or red- (665 nm) light irradiation to test which component of the composite takes part in the process or whether the SMX decomposition is a result of the charge transfer between the two moieties.

#### 3.3.1. Photocatalytic Degradation of Sulfamethoxazole in Organic Solvent

**Figure 13:**
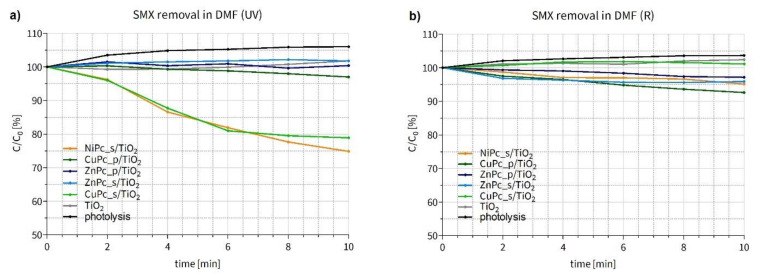
Changes in sulfamethoxazole concentration after (**a**) UV irradiation (365 nm light) (**b**) after R irradiation (665 nm light) of the DMF suspension containing a photocatalyst. The trend observed for SMX photolysis is the result of dynamic interactions between SMX molecules and DMF under irradiation conditions. Over the experiment time, SMX molecules formed associates with DMF (the absorbance values in UV-Vis measurements increased and reached plateau).

During the SMX photodegradation experiments in *N,N*-dimethylformamide under UV light (Figure 13a), two composites, namely NiPc_s/TiO_2_ and CuPc_s/TiO_2_, turned out to be the most effective, leading to 25 and 21% of SMX removal within 10 min, respectively. These two materials displayed higher activity than other Pc-grafted TiO_2_ nanoparticles obtained. A slight reduction in SMX content can also be noticed for CuPc_p/TiO_2_. Surprisingly, the use of both zinc(II) Pcs, modified with either sulfonyl or pyridyl groups, showed no significant changes in SMX concentration. Unmodified TiO_2_ also displayed no activity due to the low concentration and quenching of hydroxyl radicals in DMF [73]. We suppose that the photocatalytic mechanism occurs via type II reaction, namely energy transfer mechanism, involving the formation of singlet oxygen and organic radicals [17,74].

During the experiment conducted under red irradiation (Figure 13b), a slight reduction of the SMX concentration was observed in the experiments conducted under R light with CuPc_p/TiO_2_, ZnPc_s/TiO_2_, ZnPc_p/TiO_2_ and NiPc_s/TiO_2_. Nevertheless, none of the materials was distinctively more active than the others. As mentioned before, bare titanium dioxide is generally not expected to be activated with visible light and to enable decomposition of the target substance in DMF. Interestingly, the effectiveness of CuPc_p/TiO_2_ was even slightly higher under R than UV irradiation. Moreover, in contrast to the results shown in Figure 13a, CuPc_s/TiO_2_ displayed no visible activity in the red light.

To correctly compare the results obtained in both experiments, a few aspects should be taken into account. First, the light power was equal in all experiments (20 mW/cm^2^). Although, the number of photons was higher in the case of R irradiation (6.69 × 10^20^ m^−2^s^−1^) than UV irradiation (3.67 × 10^20^ m^−2^s^−1^), the photon energy of red light (5.45 × 10^−19^ J) is lower compared to the ultraviolet wavelength ranges (2.99 × 10^−19^ J). Second, UV light is absorbed by both parts of the composite, TiO_2_ and the phthalocyanine (in the Soret band), while red light—only by the Pc (in the Q band). Third, the photocatalyst-to-SMX ratio was low (4:1), and the phthalocyanine content constituted 1% (*w*/*w*) of each composite. Taking all of this into account, the slight, but progressing, reduction of the SMX content during the experiment under red irradiation, especially using CuPc_p/TiO_2_, can be considered a promising result. To facilitate the quantitative comparison of the degradation rates mediated by the photocatalysts, reaction constants of the studied processes were calculated for the best two materials (Table 6).

Furthermore, to better compare the performance of our photocatalytic materials in the SMX photodegradation tests under UV irradiation, we have normalized the results of the experiment by the molar quantity of deposited Pc. In fact, as the Pc content in each composite is 1% (*w*/*w*), and there are considerable differences between the molar mass values of the Pcs, the photodegradation results can be reconsidered. ΔC normalized values presented in Table 7 confirm NiPc_s/TiO_2_ and CuPc_s/TiO_2_ being the most efficient photocatalysts in SMX degradation and show that NiPc_s/TiO_2_ is, in fact, 44% better than CuPc_s/TiO_2_, even though in Figure 13a NiPc_s/TiO_2_ and CuPc_s/TiO_2_ seem to have similar effectiveness. All the calculations are available in the Supporting Information (Table S3).

#### 3.3.2. Photocatalytic Degradation of Sulfamethoxazole in Water

**Figure 14:**
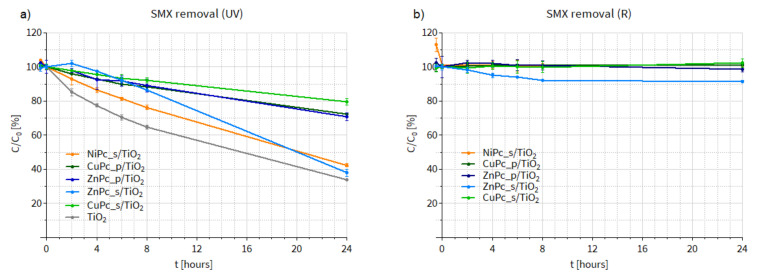
Changes in sulfamethoxazole concentration after (**a**) UV irradiation (365 nm light) (**b**) after R irradiation (665 nm light) of the water suspension containing a photocatalyst.

Having assessed the activity of our photocatalysts in DMF, we conducted SMX photodegradation tests in water (Figure 14). In this case, the SMX removal process occurred more slowly. Under UV irradiation (Figure 14a), NiPc_s/TiO_2_ was again the most active material among all the Pc-grafted TiO_2_ composites. Nevertheless, none of the materials was more efficient than the bare TiO_2_. It should be added that during the experiment under UV light conducted using ZnPc_s/TiO_2_, discoloration of the photocatalytic mixture was observed, which proves that a photobleaching effect occurred. This phenomenon consists in the degradation of the organic dye due to the attack of ^1^O_2_ on the macrocyclic ring. Alternatively, the discoloration might be a result of ZnPc_s deposited on the surface of TiO_2_ being more susceptible to the attack of hydroxyl radicals than SMX or alternatively of easier access of ROS to the anchored Pc than to SMX in solution. Therefore, the final efficiency of ZnPc_s/TiO_2_ in SMX removal under UV light was similar to that of the bare TiO_2_, because after the photobleaching occurred and the Pc was degraded, TiO_2_ was exposed to the UV light.

Noteworthy, when comparing the SMX degradation results available in the literature, several factors should be taken into account, for example, the power of the irradiation source. Nowadays, most lab-scale experiments are conducted using high-power lamps, which allow researchers to obtain high removal efficiencies. However, these conditions are difficult (and uneconomic) to implement on a large scale. In the present study, low-power LED lamps were used and, consequently, lower, yet more realistic, degradation rates were noted.

During the experiment under R irradiation, shown in Figure 14b, no changes in SMX concentration were observed when other materials (NiPc_s/TiO_2_, ZnPc_p/TiO_2_, CuPc_s/TiO_2_ CuPc_p/TiO_2_) were used. Only the use of ZnPc_s/TiO_2_ led to 10% SMX removal. In this case, the discoloration of the photocatalytic suspension was less distinct, probably due to the lower energy of the red irradiation, compared to UV light. However, Zn(II) Pcs generally display moderate to high ^1^O_2_ quantum yields (in DMF, DMSO, water and methanol) [17], so the fact that the 10% SMX removal occurred within the first 8 h of the experiment and the curve for ZnPc_s/TiO_2_ reached a plateau may be a sign of the Pc degradation.

Having this in mind, the results from the experiment in DMF, presented in the previous section, should be reconsidered. Figure 13b shows a similar trend for the ZnPc_s/TiO_2_: first, relatively rapid SMX degradation (0–6 min), followed by almost no changes in the drug concentration for the next 4 min until the end of the experiment. In the case of the photocatalytic tests in DMF, due to the low photocatalyst-to-SMX ratio, the discoloration of the suspension was practically impossible to notice. Therefore, we suppose that the photobleaching effect in the case of ZnPc_s/TiO_2_ might have also taken place during the experiments in DMF under UV-irradiation. It seems possible that this material displayed high activity, which resulted in the degradation of the dye instead of SMX. The calculated rate constants for the SMX degradation in water under UV light are presented in Table 8.

**Figure 15:**
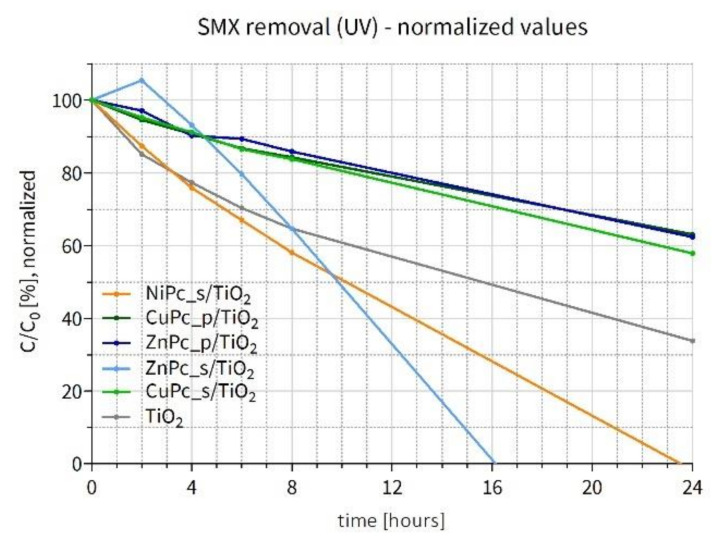
Changes in sulfamethoxazole concentration after UV irradiation (365 nm light) of the water suspension containing a photocatalyst–normalized results.

Similar to the previous section, the SMX concentration values measured throughout the experiment under UV irradiation in water were also normalized to better compare the influence of the Pcs deposited on TiO_2_. However, in this case, the results obtained in the SMX degradation experiment are normalized by the available surface area of TiO_2_ (not occupied by the Pc content). As shown in Figure 14a, none of the Pc-grafted materials turned out to be more active than the bare TiO_2_. Therefore, the following hypothesis can be put forward: if a Pc covers with TiO_2_ its entire surface, it occupies a certain part of the titania’s active surface area, blocking the incident UV light and hampering photoexcitation. Taking into account the actual Pc content on TiO_2_, obtained thanks to the TG-DSC analyses, and the TiO_2_ surface area calculated using the t-plot results, the % coverage of the photocatalyst surface was calculated (Table S2), and the remaining available surface was used to normalize the results. The obtained values are presented in Figure 15. It is apparent that NiPc_s/TiO_2_ stands out as the best material (apart from ZnPc_s/TiO_2_, which underwent photobleaching). Interestingly, the SMX degradation curve for this material lies below the curve for the bare TiO_2_, which indicates that the presence of NiPc_s is favorable for the photocatalytic reaction, given the same photocatalytically active surface area. All the calculated values are available in the Supporting Information (Table S4).

In this project, the photocatalytic composites were used in bulk form, suspended in the reaction mixture. After the experiments, they were easily removed by centrifugation and filtration, as they tend to form agglomerates in a liquid medium. Although using bulk materials allows for reaching higher reaction rates, thanks to a larger photocatalytically active surface available [75], on an industrial scale, it would require additional separation techniques to remove the photocatalysts from a slurry reactor. To avoid this problem, the photocatalysts could be immobilized, for example, incorporated into a film [9]. Such an approach allows us to eliminate leaching and, consequently, the safety concerns towards TiO_2_ applied in photocatalytic processes [76].

The present study focused on describing the relationship between the physicochemical properties and the activity of the Pc-TiO_2_ photocatalytic composites. However, to successfully implement these materials in the industrial-environmental practice, further studies should confirm their long-time stability, ex. in cycle testing. Nevertheless, in the case of the most active composites, such as ZnPc_s/TiO_2_, longer irradiation led to the photobleaching of the phthalocyanine. This phenomenon was associated with the lack of energy transfer from Pcs to TiO_2_, which could result in the production of singlet oxygen by Pcs. Singlet oxygen is a ROS characterized by a short half-life, especially in water. This suggests it would react with the Pc and not reach SMX molecules (alternatively, the phthalocyanine blocking the access of SMX to TiO_2_ would be exposed to the hydroxyl radicals produced by TiO_2_, which in turn would cause its degradation instead of SMX).

Although the presented Pc-TiO_2_ composites materials absorb visible light, they did not show any photoactivity in the red light region. Nevertheless, further attempts to improve their photocatalytic activity, such as testing under irradiation of other wavelength region or applying another preparation procedure, could be an interesting continuation of the present study.

## 4. Conclusions

Herein, new photocatalytic composites of TiO_2_ and peripherally modified phthalocyanines were fabricated by chemical deposition. The presented preparation procedure is simple and efficient, as it does not require multiple steps or high energy consumption and allows for complete deposition of the phthalocyanine on the titania surface. The materials were characterized using UV-diffuse reflectance spectroscopy, hydrodynamic particle-size analysis (*NanoSight*), surface-area analysis (N_2_ adsorption-desorption method) and thermogravimetry with differential scanning calorimetry. The UV-DRS measurements showed that the peripheral modification of the macrocyclic compounds deposited on TiO_2_ results in lower band-gap energy values (calculated for direct and indirect transition modes) than bare TiO_2_ or unmodified zinc(II) phthalocyanine-TiO_2_ composite. Particle-size analysis demonstrated that in water, the composites tend to agglomerate, as their hydrodynamic diameter is much higher than the geometric diameter of the commercial nanosized TiO_2_ P25. Surface-area analysis using the nitrogen adsorption-desorption method indicated that although the tested materials are non-porous, mesopores are formed between the particles in agglomerates. The hysteresis loops, associated with capillary condensation in mesopores, indicated that for the composites containing peripherally-modified Pc, there were more contact points between the particles in agglomerates when compared to unmodified ZnPc/TiO_2_ and bare TiO_2_. 

Furthermore, the Pc/TiO_2_ composites were applied in the photocatalytic degradation of sulfamethoxazole (SMX), an antibacterial drug. During the SMX removal experiments in an organic medium under UV irradiation, two composites were significantly more effective than the neat TiO_2_: NiPc_s/TiO_2_ and CuPc_s/TiO_2_. Under visible irradiation in the red region of the spectrum four materials led to higher SMX photodecomposition CuPc_p/TiO_2_, NiPc_s/TiO_2_, ZnPc_s/TiO_2_ and ZnPc_p/TiO_2_. The SMX photocatalytic degradation tests in water showed that only one composite, namely NiPc_s/TiO_2_, led to comparable SMX removal as the bare TiO_2_.

The physicochemical characteristics of phthalocyanine-modified TiO_2_ nanoparticles collected in this paper prove that photosensitization with organic dyes is a promising strategy to obtain a highly active and stable photocatalyst. Nevertheless, implementation of the described materials requires further efforts to enhance the performance of the composites in photocatalytic degradation of emerging contaminants.

## Figures and Tables

**Table 1:** Modifications to the chemical deposition procedure, specified for each of the prepared materials. n—calculated number of moles of the Pc used.

Pc	Formula	Formula Weight	n [mmol]	Solvent Used	Solvent Removal	pH Adjustment
**NiPc_s**	Na_4_C_32_H_12_N_8_NiO_12_S_4_	979.39	0.010	deionized water	centrifugation and washing with ethanol	no
**CuPc_p**	C_28_H_12_CuN_12_	580.02	0.017			yes, to 3–4 ^1^
**H_2_Pc_p**	C_28_H_16_CuN_12_	520.51	0.019			
**ZnPc_p**	C_28_H_12_N_12_Zn	581.88	0.017			
**ZnPc_s**	Na_4_C_32_H_12_N_8_ZnO_12_S_4_	898.18	0.011			
**CuPc_s**	Na_4_C_32_H_12_CuN_8_O_12_S_4_	984.25	0.010			
**ZnPc**	C_32_H_16_N_8_Zn	577.93	0.017	analytical grade dichloromethane	using a rotary evaporator	no

^1^ pH was adjusted with HCl and NaOH.

**Table 2:** Hydrodynamic diameter values of the particles obtained using NTA.

Photocatalytic Material	Mean Hydrodynamic Diameter [nm]	SD [nm]	PDI
TiO_2_	240	95	0.16
NiPc_s/TiO_2_	236	105	0.20
CuPc_p/TiO_2_	191	74	0.15
ZnPc_p/TiO_2_	243	182	0.56
ZnPc_s/TiO_2_	216	146	0.46
CuPc_s/TiO_2_	187	74	0.16
ZnPc/TiO_2_	250	98	0.15
TiO_2_	240	95	0.16

SD—standard deviation, PDI—polydispersity index, calculated according to the formula PDI = (SD/mean diameter)^2^.

**Table 3:** Results of the surface area analysis using the N_2_ adsorption-desorption method. S_BET_ is the specific surface area calculated using the multilayer theory of Brunauer–Emmett–Teller and S_EXT_ is the external specific surface calculated by the t-plot method.

Photocatalytic Material	S_BET_ [m^2^/g]	S_EXT_ [m^2^/g]	t-Plot Micropore Volume [cm^3^/g]	D [nm]
TiO_2_	57	55	0.000178	25.4
NiPc_s/TiO_2_	54	50	0.001488	28.2
CuPc_p/TiO_2_	52	56	−0.002796	25.2
ZnPc_p/TiO_2_	55	54	−0.000191	26.1
ZnPc_s/TiO_2_	52	52	−0.000390	27.3
CuPc_s/TiO_2_	53	53	−0.000993	26.4
ZnPc/TiO_2_	56	58	−0.001720	24.3

**Table 4:** Results of the TG-DSC analysis for each photocatalytic composite. The mass loss temperature ranges were determined based on the data shown in Figure S6.

Photocatalytic Material	Temperature Range [°C] of the Mass Loss		Mass Loss [%]	Sample Mass [mg]	Mass Loss [mg]
	Onset	Offset			
TiO_2_	20	340	1.9	10.3	0.19
NiPc_s/TiO_2_	302	467	1.0	40.1	0.40
CuPc_p/TiO_2_	200	306	1.0	59.2	0.59
	306	450			
ZnPc_p/TiO_2_	167	255	0.9	48.5	0.44
	305	460			
ZnPc_s/TiO_2_	301	480	1.3	50.9	0.66
CuPc_s/TiO_2_	199	239	1.2	60.6	0.73
	305	443			
ZnPc/TiO_2_	309	458	0.9	38.8	0.35

**Table 5:** Comparison of the heat values needed for the combustion of the neat phthalocyanines (ZnPc_p, ZnPc_s, ZnPc) and in the photocatalytic composite with TiO_2_ (ZnPc_p/TiO_2_, ZnPc_s/TiO_2_, ZnPc/TiO_2_).

Photocatalytic Material	m_s_ [mg]	Q [J/g]	Q • m_s_ [J]	Δm [mg]	Q_Δm_ [J/g]
ZnPc_p/TiO_2_	48.5	112.33	5.45	0.54	10,033
ZnPc_p	13.0	-	-	-	13,000
ZnPc_s/TiO_2_	50.9	189.19	9.64	0.78	12,349
ZnPc_s	13.8	-	-	-	9778
ZnPc/TiO_2_	38.8	214.05	8.31	0.51	16,285
ZnPc	15.9	-	-	-	13,000

m_s_–mass of the sample, Q—heat corresponding to the combustion of the Pc content deposited on TiO_2_ (calculated per gram of the photocatalytic composite), Δm—mass change in the temperature range corresponding to the combustion of the Pc, Q_Δm_—heat needed for the combustion of the Pc (calculated per gram of the Pc).

**Table 6:** The SMX degradation reaction rate constants (first-order reaction model) under UV irradiation with Pc-grafted TiO_2_ composites in DMF (Figure S9).

Photocatalytic Material	k [s^−1^]	R
NiPc_s/TiO_2_	5.13 × 10^−4^	0.996
CuPc_s/TiO_2_	4.60 × 10^−4^	0.988

**Table 7:** ΔC normalized values, calculated from relative SMX concentrations during the degradation experiment in DMF.

	NiPc_s/TiO_2_	CuPc_p/TiO_2_	ZnPc_p/TiO_2_	ZnPc_s/TiO_2_	CuPc_s/TiO_2_
ΔC, normalized	2.24	0.17	−0.03	−0.11	1.56

**Table 8:** The SMX degradation reaction rate constants (first-order reaction model) under UV irradiation with Pc-grafted TiO_2_ composites in water Figure S10.

Photocatalytic Material	k [s^−1^]	R
TiO_2_	1.32 × 10^−5^	0.998
NiPc_s/TiO_2_	9.89 × 10^−6^	0.999
ZnPc_p/TiO_2_	4.03 × 10^−6^	0.994

The regression parameters for ZnPc_s/TiO_2_ were not calculated due to the observed photobleaching effect during the experiment.

## Data Availability

All the data obtained in this study is presented either in the manuscript or in the Supplementary Materials.

## Supplementary Materials

The following supporting information can be downloaded at: https://www.mdpi.com/article/10.3390/nano12193279/s1, Scheme S1: Synthesis scheme of 4,4′,4′′,4′′′-tetraaza-29*H*,31*H*-phthalocyanine; Scheme S2: Synthesis scheme of zinc(II) 4,4′,4′′,4′′′-tetraaza-29*H*,31*H*-phthalocyanine; Scheme S3: Synthesis scheme of zinc(II) phthalocyanine-tetrasulfonic acid; Figure S1: Distribution of HiPyr species; Figure S2: Normalized Kubelka–Munk function for the tested photocatalysts; Figure S3. Band gap determination for the prepared nanomaterials—direct transition; Figure S4: Band gap determination for the prepared nanomaterials—indirect transition; Table S1: Trend line equation, correlation coefficient (R) and band gap energy values of each photocatalytic material calculated from DRS measurements using the Kubelka–Munk equation and the Tauc plot method: a) direct transition, b) indirect transition; Figure S5: t-plots determined using Harkins and Jura statistical thickness equation on the adsorption curve (N_2_ at 77 K); Figure S6: TG-DSC of prepared photocatalytic composites of phthalocyanines and TiO_2_ nanoparticles (TG and dTG data); Figure S7: TG-DSC of neat phthalocyanines (TG and HeatFlow data); Figure S8: TG-DSC of neat phthalocyanines (TG and dTG data); Figure S8: TG-DSC of neat phthalocyanines (TG and dTG data); Table S2: Percentage coverage determination from TG-DSC and N_2_ adsorption.; Figure S9: Comparison of SMX degradation rate constants (k, s^−1^) under UV irradiation with different photocatalysts; Table S3: Relative concentrations of SMX during the degradation experiment in DMF and ΔC normalized values; Figure S10: Comparison of SMX degradation rate constants (k, s^−1^) under UV irradiation with different photocatalysts; Table S4: SMX photodegradation values normalized by the TiO_2_ surface available (100% considered available for neat TiO_2_).
