# Supplementary material for: Nanocomposites of Titanium Dioxide and Peripherally Substituted Phthalocyanines for the Photocatalytic Degradation of Sulfamethoxazole
Source: Nanomaterials (Basel). 2022 Sep 21;12(19):3279. doi: 10.3390/nano12193279 (PMC9565719; doi:10.3390/nano12193279)
Supplement: Supplementary file 1 [file nanomaterials-12-03279-s001.zip › nanomaterials-1921625-supplementary.pdf]

Supporting information for

Nanocomposites of titanium dioxide and peripherally substituted phthalocyanines for the photocatalytic degradation of sulfamethoxazole

Joanna Musial[1*], Artium Belet[2], Dariusz T. Mlynarczyk[3], Michał Kryjewski[4], Tomasz Goslinski[3*], Stéphanie Lambert[2], Dirk Poelman[5], Beata J. Stanisz[1*]

1) Chair and Department of Pharmaceutical Chemistry, Faculty of Pharmacy, Poznan University of Medical Sciences, Grunwaldzka 6, 60-780 Poznań, Poland
2) Department of Chemical Engineering – Nanomaterials, Catalysis, Electrochemistry, University of Liege, Building B6a, Allée du 6 Août 11, B-4000 Liège, Belgium
3) Chair and Department of Chemical Technology of Drugs, Faculty of Pharmacy, Poznan University of Medical Sciences, Grunwaldzka 6, 60-780 Poznań, Poland
4) Chair and Department of Inorganic and Analytical Chemistry, Faculty of Pharmacy, Poznan University of Medical Sciences, Rokietnicka 3, 60-806 Poznań, Poland
5) LumiLab, Department of Solid State Sciences, Ghent University, Krijgslaan 281 S1, 9000 Ghent, Belgium

* Correspondence: joanna.musial@ump.edu.pl (J.M.); tomasz.goslinski@ump.edu.pl (T.G); bstanisz@ump.edu.pl (B.J.S.)

Table of Contents

1. Synthesis of phthalocyanines 2
 1.1. Synthesis of 4,4′,4′′,4′′′-tetraaza-29H,31H-phthalocyanine (**2HPc_p**) 2
 1.2. Synthesis of zinc(II) 4,4′,4′′,4′′′-tetraaza-29*H*,31*H*-phthalocyanine (**ZnPc_p**) 2
 1.3. Synthesis of zinc(II) phthalocyanine tetrasulfonic acid (**ZnPc_s**) 3
2. Preparation of photocatalytic materials 3
3. UV-Vis diffuse reflectance spectroscopy (DRS) – bandgap determination 4
4. Surface area analysis 8
5. TG-DSC 9
6. Photochemical studies 11
 6.1. Photocatalytic degradation of sulfamethoxazole in DMF 11
 6.2. Photocatalytic degradation of sulfamethoxazole in water 12
7. References 13

1. **Synthesis of phthalocyanines**

The identity of the isolated products was confirmed by comparison with the literature data.

1.1. Synthesis of 4,4′,4′′,4′′′-tetraaza-29H,31H-phthalocyanine (**2HPc_p**)

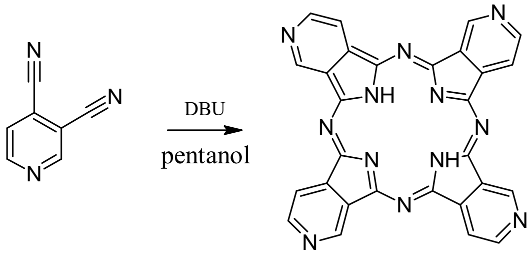

Scheme S1. Synthesis scheme of 4,4′,4′′,4′′′-tetraaza-29*H*,31*H*-phthalocyanine.

The literature procedure was adapted [1].

3,4-Dicyanopyridine (200 mg, 1.550 mmol), 2.0 mL of pentanol, and 1,8-diazabicyclo[5.4.0]undec-7-ene (DBU; 10 µL, 0.067 mmol) were mixed in a microwave reaction vessel. The reaction mixture was heated in a microwave reactor to 200°C and stirred at this temperature for 30 minutes. After cooling down to room temperature, the solvent was evaporated. The dry residue was filtered and washed with *n*-hexane and then acetone until discoloration of the filtrate was observed. The product was left to dry for 20 hours.

Yield 84 mg (42%).

1.2. Synthesis of zinc(II) 4,4′,4′′,4′′′-tetraaza-29*H*,31*H*-phthalocyanine (**ZnPc_p**)

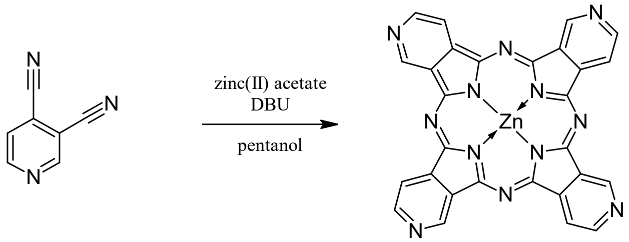

Scheme S2. Synthesis scheme of zinc(II) 4,4′,4′′,4′′′-tetraaza-29*H*,31*H*-phthalocyanine.

The literature procedure was adapted [2].

First, 200.0 mg of 3,4-pyridine dicarbonitrile and 237 mg of zinc acetate were added to a G10 vial. 3,4-Dicyanopyridine (200 mg, 1.550 mmol), 2.0 mL of pentanol, zinc(II) acetate (237 mg, 1.292 mmol), and 1,8-diazabicyclo[5.4.0]undec-7-ene (10 µL, 0.067 mmol) were mixed in a microwave reaction vessel. The reaction mixture was heated in a microwave reactor to 200°C and stirred at this temperature for 30 minutes. After cooling down to room temperature, the solvent was evaporated. The dry residue was filtered and washed in turn with *n*-hexane, acetone and methanol, with each until discoloration of the filtrate was observed. The product was left to dry for 20 hours.

Yield 101 mg (45%).

1.3. Synthesis of zinc(II) phthalocyanine tetrasulfonic acid (**ZnPc_s**)

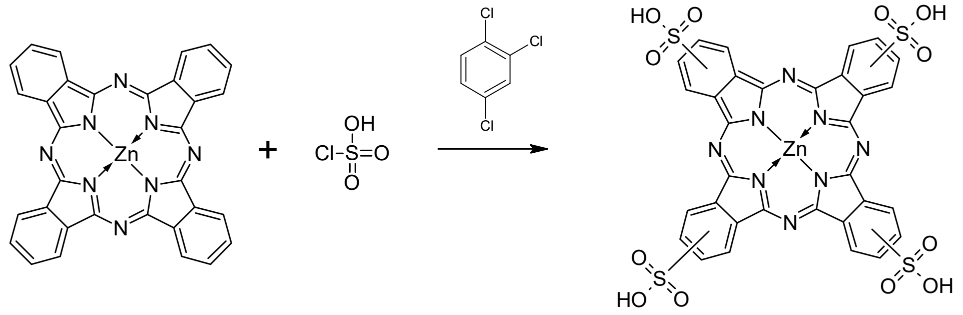

Scheme S3. Synthesis scheme of zinc(II) phthalocyanine-tetrasulfonic acid.

The preparation was based on the procedure described by Feofanov et al. [3].

Zinc(II) phthalocyanine (450 mg, 0.779 mmol), 10 mL of 1,2,4-trichlorobenzene and chlorosulfonic acid (0.3 mL, 4.513 mmol) were heated at 180°C for 2 hours under constant stirring. After cooling down to room temperature, the mixture was filtered. The dark blue solid was transferred to a beaker containing 50 mL of 1% NaOH, and stirred for 15 min. The mixture was filtered again and the dark blue filtrate was collected. 10% HCl was added to the filtrate, stirred and left for 15 min to precipitate the product. The mixture was filtered, dark green-blue precipitate was subsequently washed with ethanol and acetone and left to dry for 20h.

Yield 544 mg (71%).

2. Preparation of photocatalytic materials

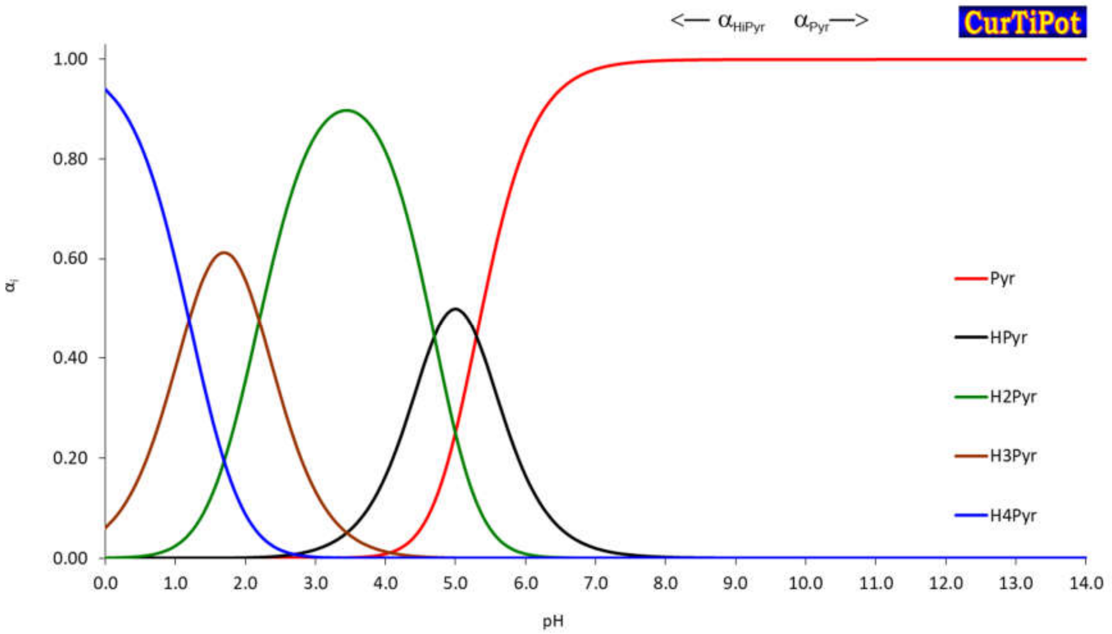

Figure S1. Distribution of HiPyr species (calculated using the CurTiPot freeware [4]).

3. UV-Vis diffuse reflectance spectroscopy (DRS) – bandgap determination

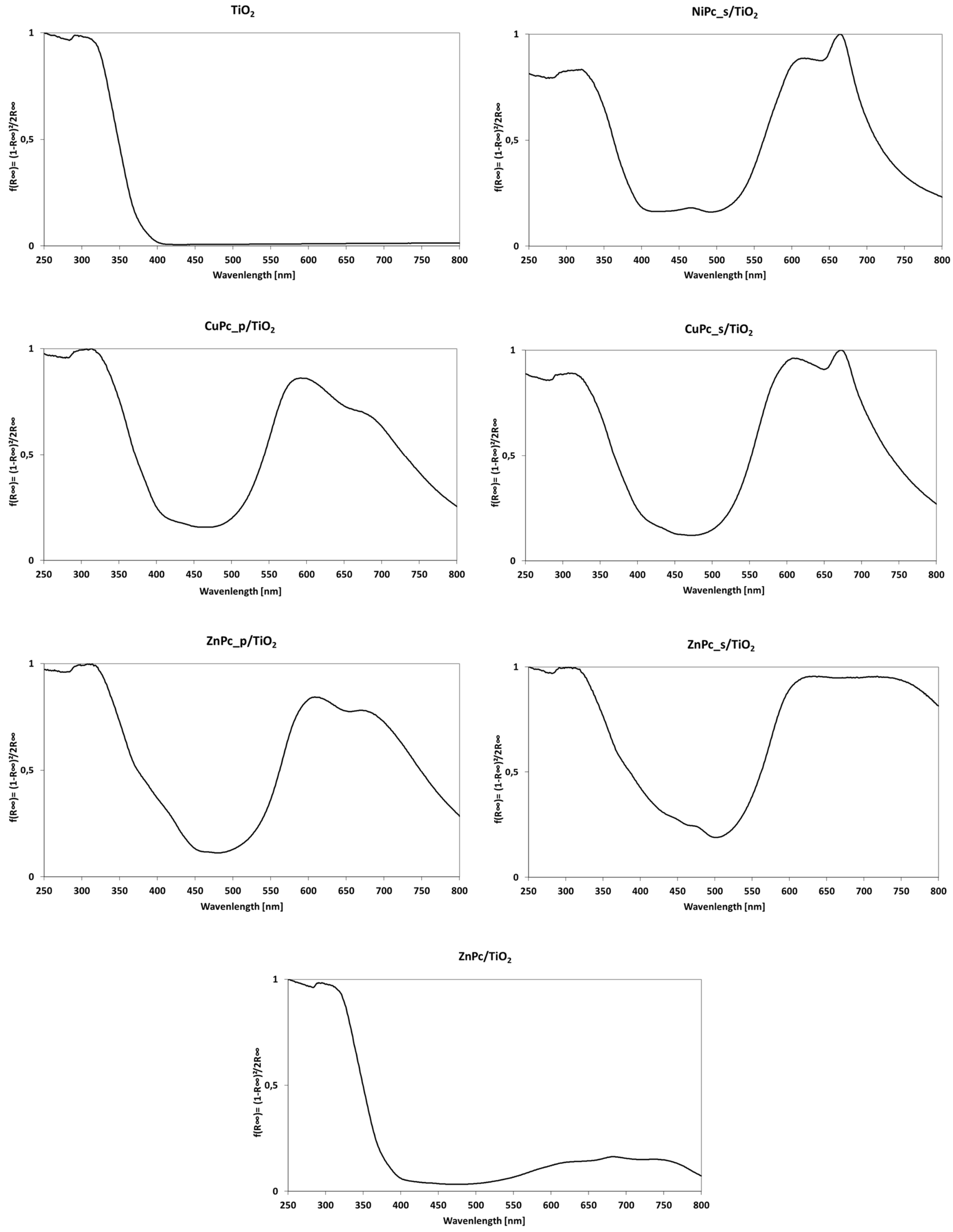

Figure S2. Normalized Kubelka-Munk function for the tested photocatalysts.

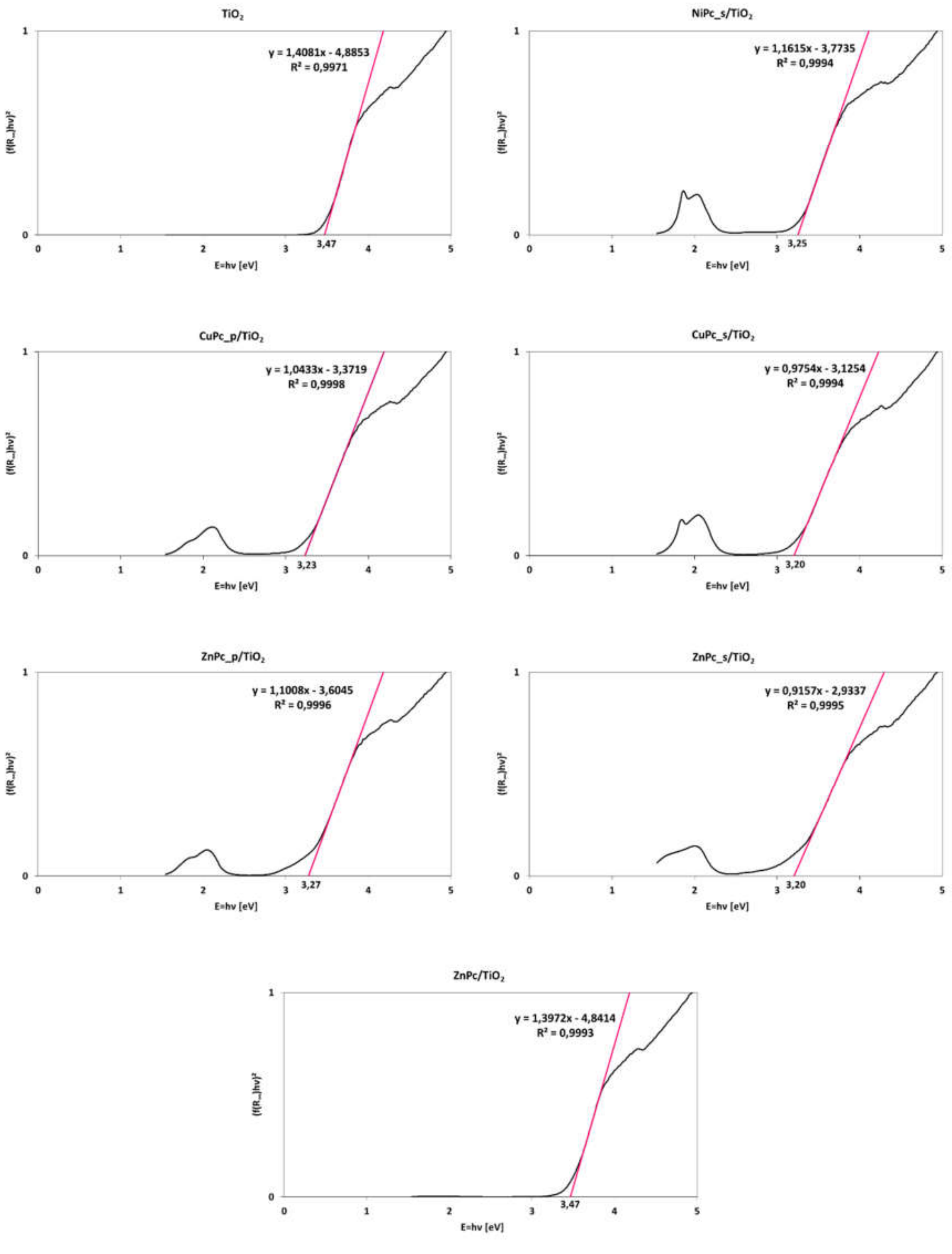

Figure S3. Band gap determination for the prepared nanomaterials – direct transition.

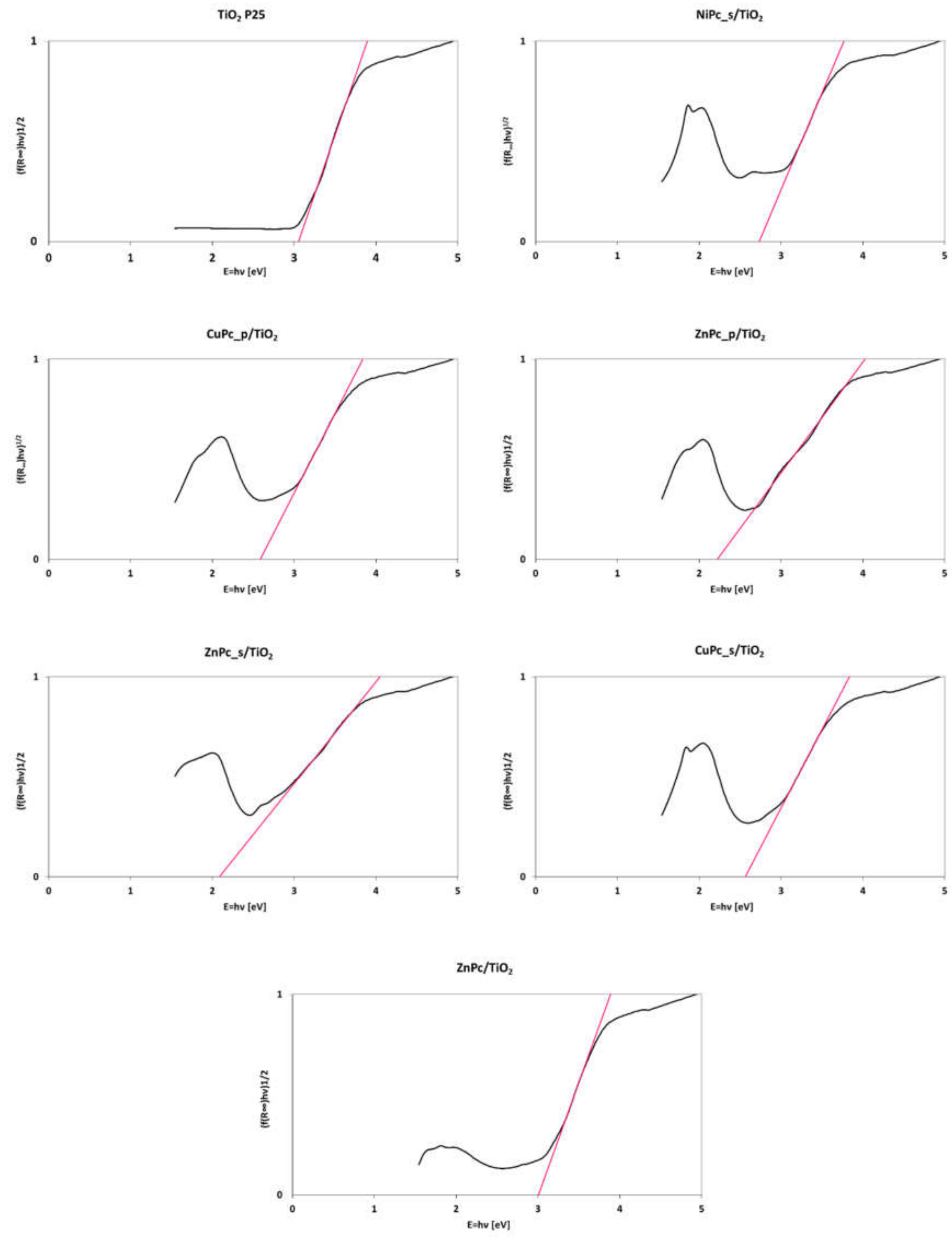

Figure S4. Band gap determination for the prepared nanomaterials – indirect transition.

Table S1. Trend line equation, correlation coefficient (R) and band gap energy values of each photocatalytic material calculated from DRS measurements using the Kubelka–Munk equation and the Tauc plot method: a) direct transition, b) indirect transition.

a)

Sample	Trendline equation	R	Direct band gap energy [eV]
TiO_2	y = 1.4081x – 4.8853	0.9985	3.47
NiPc_s/TiO_2	y = 1.1615x – 3.7735	0.9997	3.25
CuPc_p/TiO_2	y = 1.0433x – 3.3719	0.9999	3.23
ZnPc_p/TiO_2	y = 1.1008x – 3.6045	0.9998	3.27
ZnPc_s/TiO_2	y = 0.9157x – 2.9337	0.9998	3.20
CuPc_s/TiO_2	y = 0.9754x – 3.1254	0.9997	3.20
ZnPc/TiO_2	y = 1.3972x – 4.8414	0.9997	3.47

b)

Sample	Trendline equation	R	Indirect band gap energy [eV]
TiO_2	y = 1.1953x – 3.6531	0.9987	3.06
NiPc_s/TiO_2	y = 0.9676x – 2.6454	0.9997	2.73
CuPc_p/TiO_2	y = 0.7981x – 2.0646	0.9990	2.59
ZnPc_p/TiO_2	y = 0.5548x – 1.2325	0.9981	2.22
ZnPc_s/TiO_2	y = 0.5104x – 1.0662	0.9987	2.09
CuPc_s/TiO_2	y = 0.7865x – 2.0162	0.9997	2.56
ZnPc/TiO_2	y = 1.1394x – 3.4285	0.9995	3.01

4. Surface area analysis

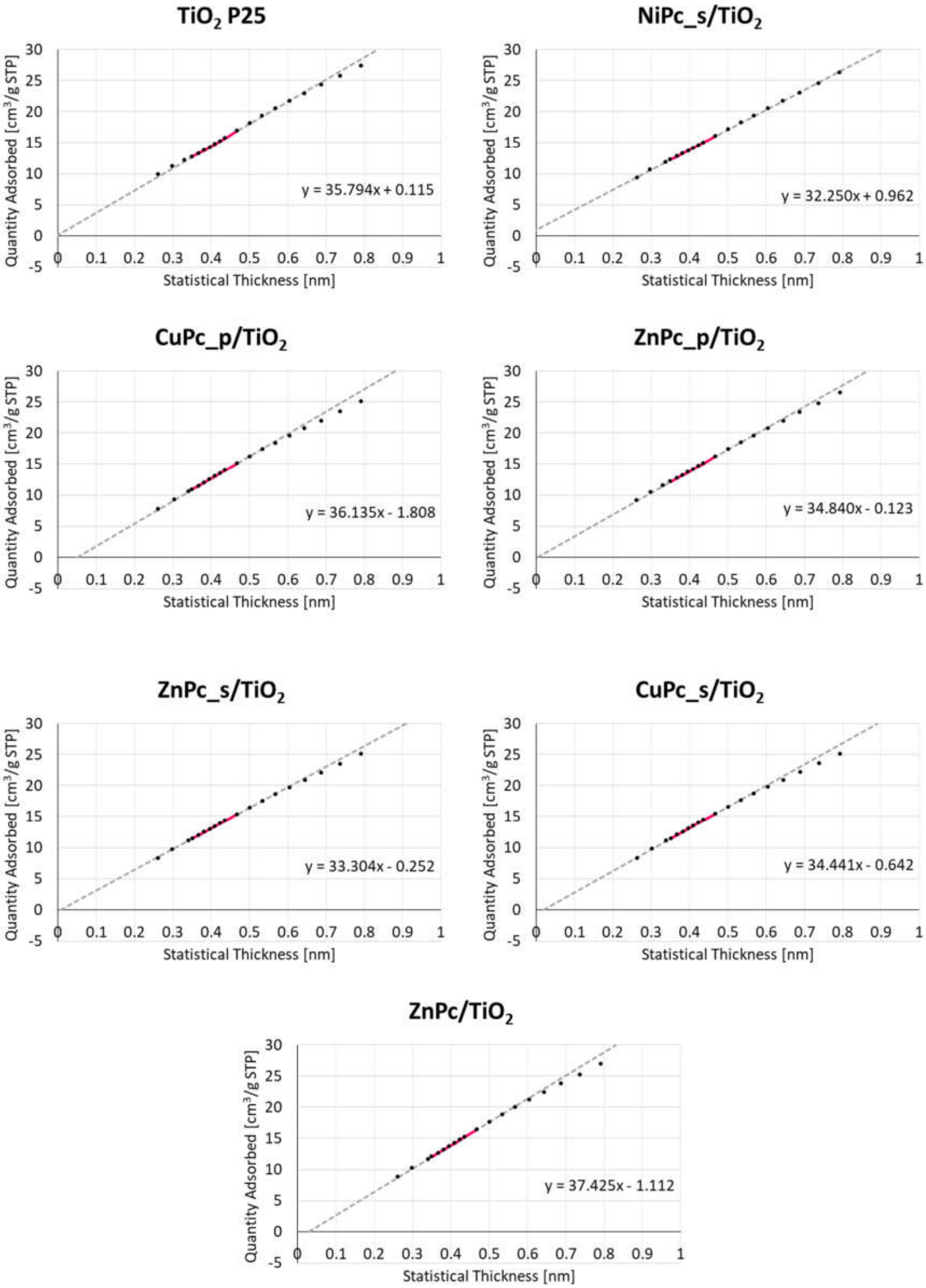

Figure S5. t-plots determined using Harkins and Jura statistical thickness equation on the adsorption curve (N_2 at 77K). Linear regression equation (-----) was calculated from fitted points (——).

5. TG-DSC

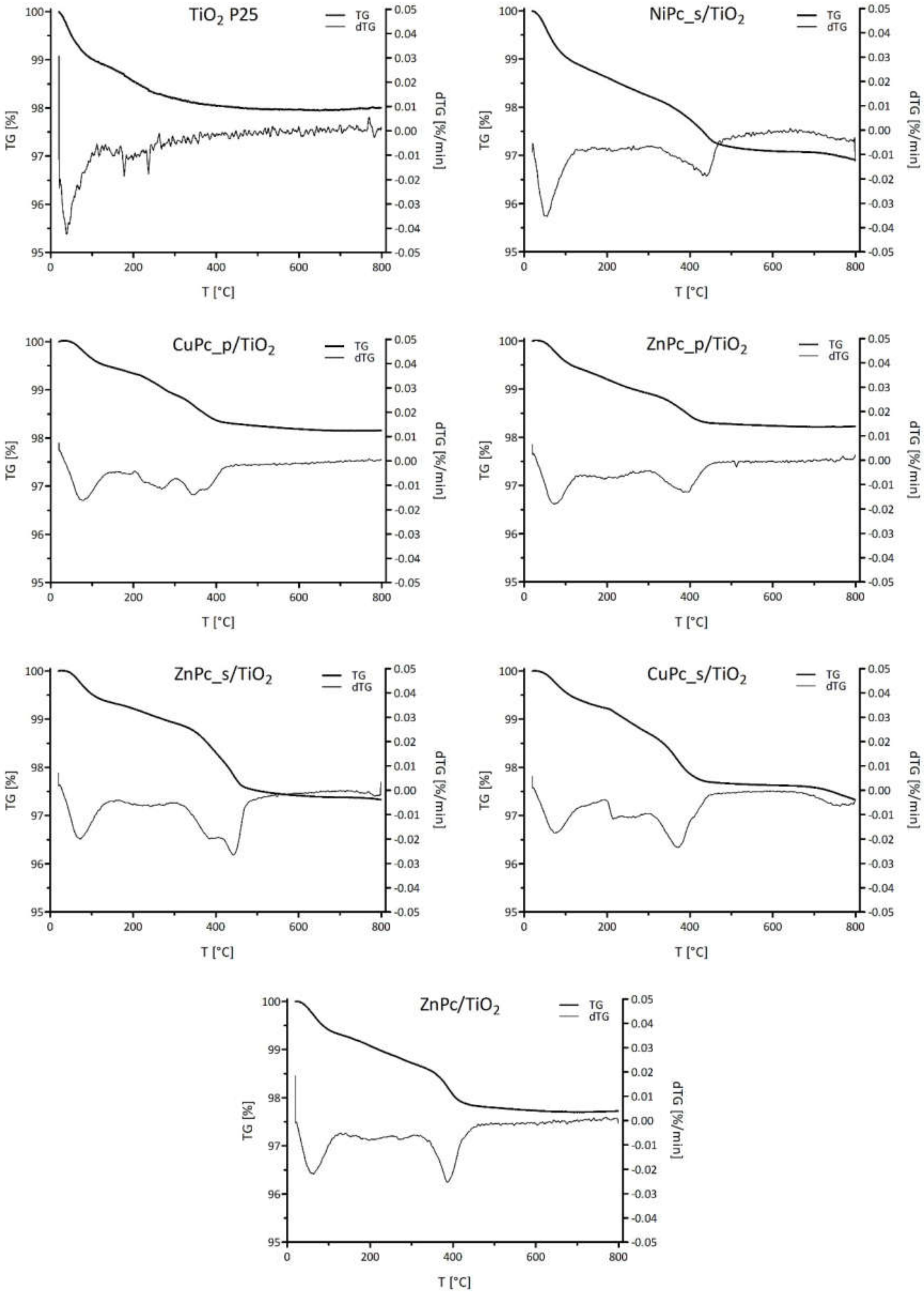

Figure S6. TG-DSC of prepared photocatalytic composites of phthalocyanines and TiO_2 nanoparticles (TG and dTG data).

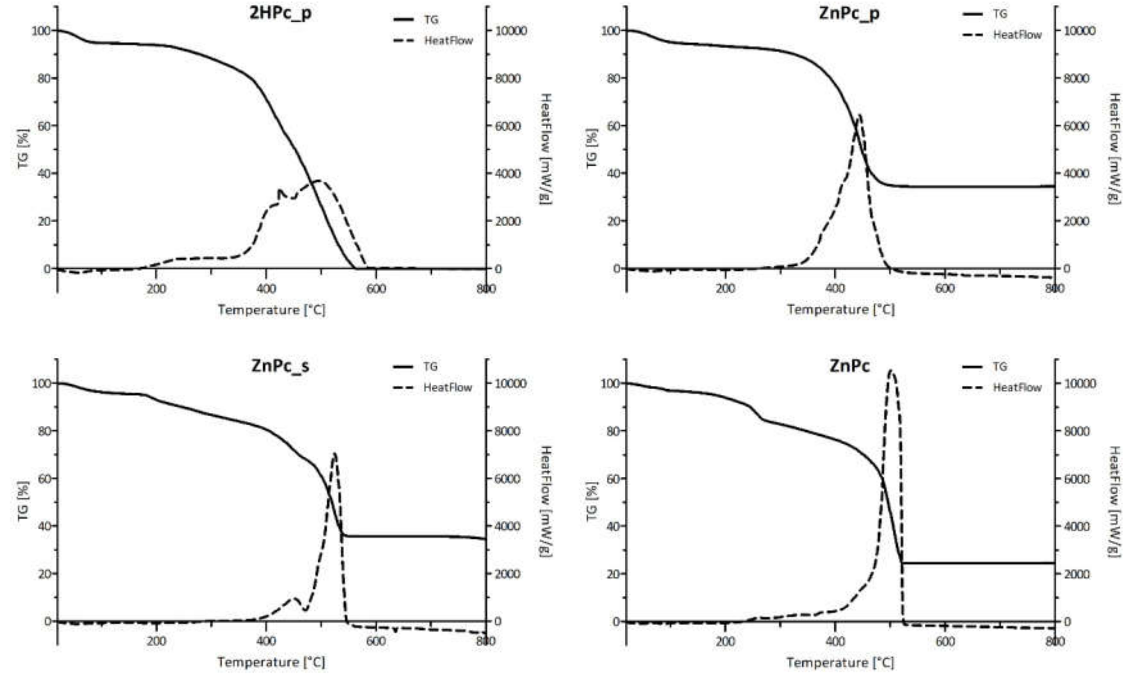

Figure S7. TG-DSC of neat phthalocyanines (TG and HeatFlow data).

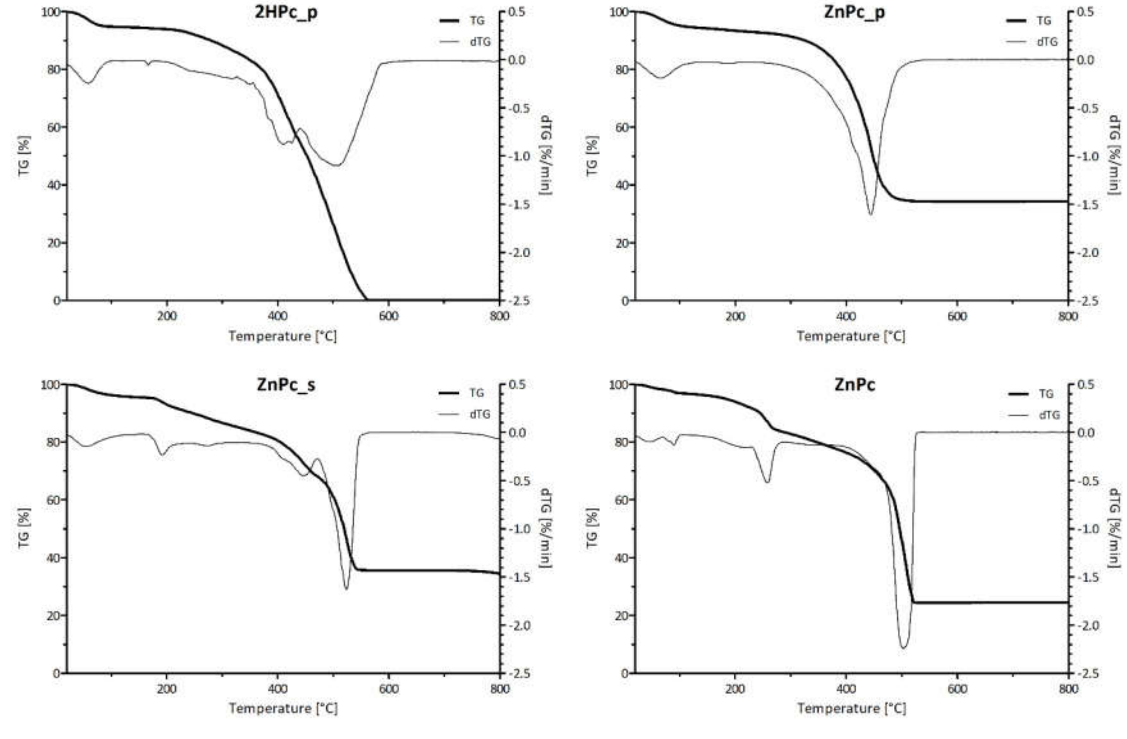

Figure S8. TG-DSC of neat phthalocyanines (TG and dTG data).

6. Photochemical studies

Table S2. Percentage coverage determination from TG-DSC and N_2 adsorption.

Photocatalytic material	Molar mass	Mass loss [mg]	$n_{Pc}/m_{Pc/TiO2}$ [µmol/g]	Surface of one Pc molecule [nm²]	Total surface of Pc [m²]	TiO_2 surface available [m²]	% coverage
**TiO_2**	79.87	0.19	-	-	-	-	-
**NiPc_s/TiO_2**	887.39	0.40	11.241	3.42	0.928	2.139	43.4
**CuPc_p/TiO_2**	580.02	0.59	17.183	1.28	0.784	3.197	24.5
**ZnPc_p/TiO_2**	581.88	0.44	15.591	1.28	0.583	2.622	22.2
**ZnPc_s/TiO_2**	806.18	0.66	16.084	3.42	1.686	2.730	61.8
**CuPc_s/TiO_2**	892.25	0.73	13.501	3.39	1.670	3.250	51.4

6.1. Photocatalytic degradation of sulfamethoxazole in DMF

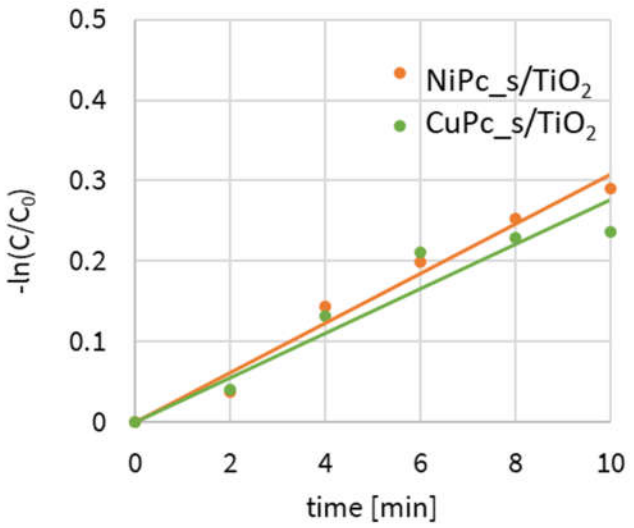

Figure S9. Comparison of SMX degradation rate constants (k, s^{-1}) under UV irradiation with different photocatalysts.

Table S3. Relative concentrations of SMX during the degradation experiment in DMF and ΔC normalized values.

	TiO_2	NiPc_s/TiO_2	CuPc_p/TiO_2	ZnPc_p/TiO_2	ZnPc_s/TiO_2	CuPc_s/TiO_2
C_0 [%]	100.00	100.00	100.00	100.00	100.00	100.00
C_{10} [%]	101.78	74.85	97.00	100.40	101.79	78.89
$\Delta C = C_0 - C_{10}$ [%]	-1.78	25.15	3.00	-0.40	-1.79	21.11
**ΔC, normalized**	**-**	**2.24**	**0.17**	**-0.03**	**-0.11**	**1.56**

where C_0 and C_{10} are the SMX concentrations after 0 and 10 min of the experiment, respectively; ΔC values were normalized by $n_{Pc}/m_{Pc/TiO2}$ [µmol/g].

6.2. Photocatalytic degradation of sulfamethoxazole in water

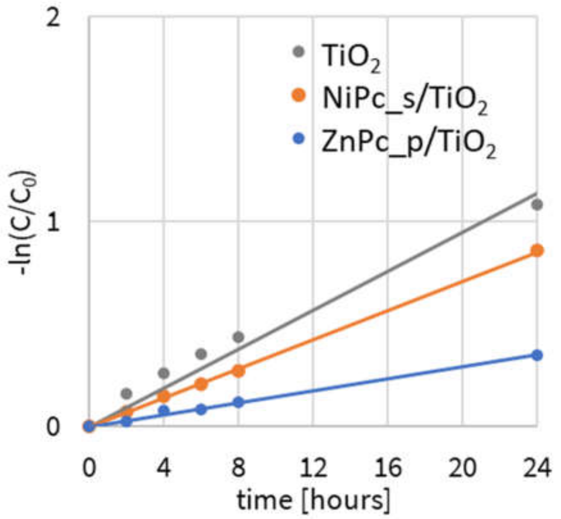

Figure S10. Comparison of SMX degradation rate constants (*k*, s^{-1}) under UV irradiation with different photocatalysts.

Table S4. SMX photodegradation values normalized by the TiO_2 surface available (100% considered available for neat TiO_2).

time [min]	**TiO_2**	**NiPc_s/TiO_2**	**CuPc_p/TiO_2**	**ZnPc_p/TiO_2**	**ZnPc_s/TiO_2**	**CuPc_s/TiO_2**
0	100.00	100.00	100.00	100.00	100.00	100.00
2	85.11	87.36	94.56	97.10	105.45	95.23
4	77.31	75.86	90.72	90.23	93.13	91.17
6	70.33	67.02	86.76	89.38	79.62	86.50
8	64.67	58.08	84.30	85.89	64.65	83.76
24	33.80	-1.82	63.12	62.36	-62.45	57.86

7. References

[1] C. Ramirez, C. Antonacci, J. Ferreira, R.D. Sheardy, The Facile Synthesis and Characterization of Novel Cationic Metallated and Nonmetallated Tetrapyridino Porphyrazines Having Different Metal Centers, Synthetic Communications. 34 (2004) 3373–3379. https://doi.org/10.1081/SCC-200030591.

[2] W.S. Szulbinski, J.R. Kincaid, Synthesis and Spectroscopic Characterization of Zinc Tetra(3,4-pyridine)Porphyrazine Entrapped within the Supercages of Y-Zeolite, Inorg. Chem. 37 (1998) 5014–5020. https://doi.org/10.1021/ic971118s.

[3] A. Feofanov, A. Grichine, T. Karmakova, N. Kazachkina, E. Pecherskih, R. Yakubovskaya, E. Luḱyanets, V. Derkacheva, M. Egret-Charlier, P. Vigny, Chelation with Metal is not Essential for Antitumor Photodynamic Activity of Sulfonated Phthalocyanines, Photochemistry and Photobiology. 75 (2007) 527–533. https://doi.org/10.1562/0031-8655(2002)0750527CWMINE2.0.CO2.

[4] I.G.R. Gutz, CurTiPot – pH and Acid–Base Titration Curves: Analysis and Simulation freeware (version 4.3.1), 2021. http://www.iq.usp.br/gutz/Curtipot_.html.
